# Sugar Reduction Initiatives in the Eastern Mediterranean Region: A Systematic Review

**DOI:** 10.3390/nu15010055

**Published:** 2022-12-22

**Authors:** Ayoub Al-Jawaldeh, Mandy Taktouk, Sally Naalbandian, Hassan Aguenaou, Nawal Al Hamad, Salima Almamary, Hend Ali Al-Tamimi, Salah Abdulla Alyafei, Rawhieh Barham, Maha Hoteit, Munawar Hussain, Hanan Massad, Lara Nasreddine

**Affiliations:** 1 Regional Office for the Eastern Mediterranean (EMRO), World Health Organization (WHO), Cairo 7608, Egypt; 2 Nutrition and Food Sciences Department, Faculty of Agriculture and Food Sciences, American University of Beirut, Beirut 11-0236, Lebanon; 3 Science and Agriculture Library, American University of Beirut, Beirut 11-0236, Lebanon; 4 Joint Research Unit in Nutrition and Food, RDC-Nutrition AFRA/IAEA, Ibn Tofail University-CNESTEN, Kenitra 14000, Morocco; 5 The Public Authority for Food and Nutrition, Kuwait City 43600, Kuwait; 6 Nutrition Department, Ministry of Health, Muscat 393, Oman; 7 Health Promotion and Non Communicable Disease (NCD) Division, Public Health Department, Ministry of Public Health, Doha 42, Qatar; 8 Nutrition Department, Ministry of Health, Amman 11118, Jordan; 9 Faculty of Public Health, Lebanese University, Beirut 6573, Lebanon; 10 PHENOL Research Group (Public Health Nutrition Program-Lebanon), Faculty of Public Health, Lebanese University, Beirut 6573, Lebanon; 11 Food Policy Program Consultant, Global Health Advocacy Incubator, Islamabad 45710, Pakistan; 12 National Consumer Protection Association, Amman 11190, Jordan

**Keywords:** total sugar, added sugar, free sugar, intake, reduction, strategy, implementation, evaluation, eastern mediterranean region

## Abstract

This systematic review aims to identify and characterize existing national sugar reduction initiatives and strategies in the Eastern Mediterranean Region. For this purpose, a systematic review of published and grey literature was performed. A comprehensive list of search terms in the title/abstract/keyword fields was used to cover the four following concepts (1) sugar, (2) reduction OR intake, (3) policy and (4) EMR countries. A total of 162 peer-reviewed documents were identified, until the 2nd of August 2022. The key characteristics of the identified national strategies/initiatives included the average sugar intake of each country’s population; sugar levels in food products/beverages; implementation strategies (taxation; elimination of subsidies; marketing regulation; reformulation; consumer education; labeling; interventions in public institution settings), as well as monitoring and evaluation of program impact. Twenty-one countries (95%) implemented at least one type of sugar reduction initiatives, the most common of which was consumer education (71%). The implemented fiscal policies included sugar subsidies’ elimination (fourteen countries; 67%) and taxation (thirteen countries 62%). Thirteen countries (62%) have implemented interventions in public institution settings, compared to twelve and ten countries that implemented food product reformulation and marketing regulation initiatives, respectively. Food labeling was the least implemented sugar reduction initiative (nine countries). Monitoring activities were conducted by four countries only and impact evaluations were identified in only Iran and Kingdom of Saudi Arabia (KSA). Further action is needed to ensure that countries of the region strengthen their regulatory capacities and compliance monitoring of sugar reduction policy actions.

## 1. Introduction

Countries of the Eastern Mediterranean Region (EMR) are witnessing the nutrition transition, with its characteristic shifts in food consumption patterns and lifestyle [1]. One of the main features of the nutrition transition is an increase in the consumption of sugar, sweetened processed foods and beverages [2,3]. This may pose a public health threat given that high sugar intakes were proposed to increase the risk of excessive weight gain and cardiometabolic diseases in the population [4,5].

Sugars may occur naturally in foods, including fruits, vegetables, and milk/dairy products, but may also be added to food items during preparation and processing [5,6]. There are different terminologies that have been proposed to discriminate between the different types of sugar. The term “Total Sugars” (TS) refers to all mono- and disaccharides that are present in a food item or beverage, whether added or naturally occurring. In contrast, the term “Added Sugars” (AS) refers only to sugars that were intentionally added to foods/beverages in order to improve palatability, impart a sweet flavor, preserve and/or confer specific functional attributes [5]. The World Health Organization (WHO) has proposed the term “Free Sugars” (FS), which comprises “monosaccharides and disaccharides added to foods and beverages by the manufacturer, cook, or consumer (i.e., AS), plus sugars naturally present in honey, syrups, fruit juices, and fruit juice concentrates (i.e., non-milk extrinsic sugars)” [7]. The FS term is thus utilized when referring to sugars that may have physiological effects that are different from those attributed to intrinsic sugars found within intact plant cell walls or naturally occurring lactose in milk [5,8].

High consumption of sugar, particularly AS and FS, has been linked to poor overall dietary quality, and excessive energy intake (EI), while contributing to energy imbalance [9,10,11,12]. Excessive sugar intakes in adults have been implicated in the epidemics of obesity, cardiovascular disorders, type 2 diabetes, non-alcoholic fatty liver diseases, and their downstream cardiometabolic abnormalities such as metabolic syndrome, dyslipidemia and chronic inflammation [13,14,15]. It was also reported that both high sugar intakes and hyperglycemia can affect the intestinal barrier, thus heightening gut permeability and leading to profound gut microbiota dysbiosis and disturbances in mucosal immunity, which in turn can increase susceptibility to infections [13]. In addition, a recent meta-analysis has reported a significant association between high sugar intake and increased risk of cognitive disorders in middle-aged and elderly populations [16]. In the pediatric and adolescent population, high intake of sugar was associated with an increased risk of overweight and obesity [17,18], dyslipidemia [19], insulin resistance [20], as well as dental caries [21].

Health agencies in the United States (US) have recommended up to 10% of total calories from AS [22]. In 2015, the WHO updated dietary guidelines pertinent to sugar intake and recommended decreasing the intake of FS to less than 10% EI as a ‘strong recommendation’ [7], and to less than 5% as a conditional recommendation [7]. The Scientific Advisory Council on Nutrition in England adopted a relatively similar approach, providing a recommendation of 10% of calories from AS, with the ultimate goal of achieving an even lower intake of 5% [23]. The American Heart Association has called for even stricter restrictions on calories from AS, with a proposed upper limit of no more than 150 kcal per day for the average adult man and no more than 100 kcal of AS per day for the average adult woman [24,25]. In 2022, the European food safety agency (EFSA), recommended that the intake of “added and free sugars should be as low as possible in the context of a nutritionally adequate diet”.

In countries of the EMR, food supply data show that the contribution of sugar (as a commodity) to capita daily food energy supply has been following a consistently increasing trend over time, with estimates of its average contribution to energy supply ranging between 9% and 15% [1,26]. At the same time, the region harbors one of the highest burdens of obesity worldwide, coupled with an alarming increase in the incidence and prevalence of diet-related noncommunicable diseases (NCDs) including cardiovascular diseases (CVDs), type 2 diabetes and certain types of cancer [1]. In 2016, the WHO Regional Office for the Eastern Mediterranean Region has set a policy goal to lower sugar intake “in order to reduce the risk of NCDs in children and adults, with a particular focus on the prevention of unhealthy weight gain and associated conditions, such as diabetes and dental caries”. The WHO EMR policy document included a set of measures for the reduction of sugar intake, including the reformulation of sugar-rich foods and drinks; setting standards for all foods and beverages served by government-sponsored institutions; restricting the promotion of sugar-enriched products, especially beverages; imposing restrictions on the marketing of sugar-enriched foods and drinks across all media platforms; adopting nutritional profiling system to set clear definitions for high sugar foods and drinks; eliminating national sugar subsidies and introducing progressive taxes initially on sweetened beverages and ultimately on all foods and drinks with AS; improving and facilitating accredited training on diet and health for individuals with opportunities to affect population food choices; and providing public education on nutrition and health. The reduction of sugar has also been included as a priority action in the WHO strategy on nutrition for the Eastern Mediterranean Region, 2020–2030 [27], and the regional framework for action on obesity prevention 2019–2023 [28]. Despite the guidance provided by the WHO EMR on sugar reduction policies, there has been no systematic appraisal of national sugar reduction efforts in various countries of the EMR. It is in this context that this systematic review was performed with the aim of identifying and documenting existing national sugar reduction strategies and initiatives in countries of the EMR, and assessing the impact of these strategies when available data allows it.

## 2. Materials and Methods

**Figure 1:**
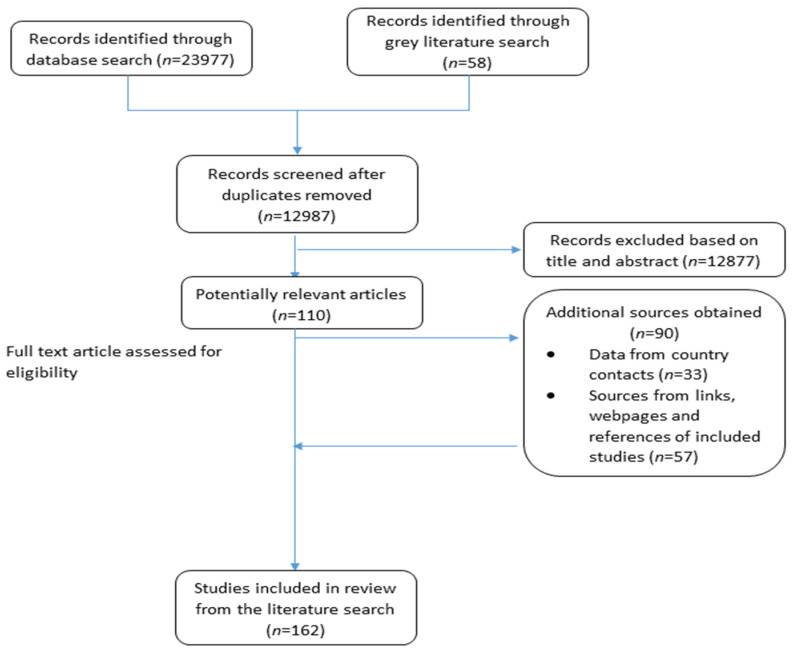
Search and identification process of potential references from the literature.

This study was written in conformity with the Preferred Reporting Items for Systematic Reviews and Meta-analyses (PRISMA) guidelines (Table S1) and was not registered in PROSPERO. The search strategy and methodology adopted in this study are comparable to the approach adopted in previous studies [29,30,31]. In brief, data related to sugar reduction strategies and initiatives were identified based on a search of peer-reviewed as well as grey literature, in addition to obtaining supplementary information by establishing direct contact with program country leaders or focal points (Figure 1).

### 2.1. Search Strategy

A search of 11 electronic databases was conducted between 24 March 2021 and 5 April 2021. These databases comprised: Directory of Open Access Journals, CAB Direct, MEDLINE (ovid), PubMed, Scopus, Web of Science Core Collections, Food Science and Technology Abstracts, Al Manhal, Arab World Research Source (AWRS), E-Marefa and Iraqi Academic Scientific Journals (IASJ). The last 4 databases are specific to the Arab region, whereby Arabic keywords were used in the search. In addition to the use of a controlled vocabulary (MeSH in PubMed and MEDLINE (ovid)), a comprehensive list of search terms was also used in the title/abstract/keyword fields to cover the four following concepts (1) sugar, (2) reduction OR intake, (3) policy and (4) EMR countries. Table A1 in Appendix A provides the detailed list of the search terms used pertaining to the four concepts while searching the various databases. In addition, an example of a database search is provided in Table S2 for MEDLINE (ovid). In the search, materials that were published in the English, Arabic or French languages were considered, and any material published prior to 1995 was excluded. Articles that were published after the initial search were identified via email alerts (up until 29 August 2022).

A search of the grey literature was also conducted, using OpenGrey, Google Scholar, the Global Database on the Implementation of Nutrition Action (GINA), the WHO EMRO (Regional Office for the Eastern Mediterranean) website as well as governmental websites (such as the websites of the Ministries of Health in the various EMR countries). This search focused on materials published post 1995 in English, Arabic or French languages. Two independent researchers (MT and LN) performed the screening of the titles, abstracts and full text articles of relevant articles, based on the inclusion and exclusion criteria described in the section below. The two researchers engaged in discussions to resolve the minor discrepancies that emanated from the two screening stages.

### 2.2. Inclusion and Exclusion Criteria

Articles were included in this systematic review if they provided information on sugar baseline assessment (including intake of sugar; levels in foods/beverages; knowledge, attitudes and behavior (KAB), or the development, implementation, monitoring/evaluation of sugar reduction initiatives at the national level).

National sugar reduction initiatives/strategies were defined as having a governmental body involved [26,32,33], in addition to one or more of the following: (1) a national action plan to reduce sugar intake at the population level [26,32]; (2) a sugar reduction/replacement program (e.g., setting limits for the levels of sugar in food products) [26,33]; (3) awareness campaigns or consumers’ education programs aimed at improving sugar-related KAB [26]; (4) labeling schemes that are specific to sugar/AS or mandatory declaration of sugar/AS on nutrition labels [26,33]; (5) taxation policies targeting high sugar foods and beverages [26,32,33]; (6) eliminating sugar subsidies [26,32]; (7) regulation of the marketing of high sugar foods/beverages [26,33]; and (8) sugar reduction initiatives in specific settings (such as schools, hospitals, workplaces, etc.) [26,33].

Studies that are based on randomized-control trial design or case-control studies, as well as those dealing with unhealthy individuals or specific population groups (such as pregnant women, individuals on therapeutic diets etc.) were excluded from the review. Individual studies/articles were also excluded from the review if they were published prior to 1995, or in any language other than English, Arabic or French.

### 2.3. Data Extraction

Data extraction was performed by two researchers (MT and LN). For the few discrepancies between the researchers, consensus was reached via discussion.

The key characteristics of each sugar reduction initiative were entered into a database that was developed by the researchers to examine baseline assessments (population TS intake; population AS intake; population FS intake; sugar levels in food products/beverages, sugar-related KAB), leadership and strategic approach, implementation strategies (taxation; elimination of subsidies; regulation on the marketing of high-sugar foods and beverages; product reformulation; consumer education; food labeling; interventions in public institution settings), monitoring (population intake, levels in food products/beverages, KAB), and evaluation of program impact [26,32,33].

### 2.4. Seeking Supplementary Information

A questionnaire was developed based on relevant literature [34,35] and was shared with country experts or program leaders in various countries of the EMR to confirm and obtain supplementary data, if available. Country experts or program leaders were invited to complete the questionnaire and/or share it with their local contacts to seek additional information and details. Accordingly, the database was updated with any additional data.

### 2.5. Analysis

The key characteristics of each of the identified national sugar reduction strategy were entered into the database, according to the pre-developed framework that includes baseline assessments; leadership/strategic approach; implementation strategies; data monitoring and evaluation of program impact. Countries were then classified as ‘having a developed strategy’ for sugar reduction, ‘having a planned strategy’ or ‘having no strategy’. Strategies were considered to be “planned” if the sugar reduction initiatives were still under development, or if an action plan had already been established but without evidence of implementation. The proportion of countries reporting on each of the key characteristic were determined, and expressed as percentages.

## 3. Results

### 3.1. Search Results

In total, 162 peer-reviewed articles, grey literature documents, websites and references from country contacts were identified via the literature search. Of these, 72 were peer-reviewed relevant articles, and 90 were additional documents/sources obtained from country contacts (through the questionnaires), webpages, links, and references from within the included articles (Figure 1).

### 3.2. Assessment of Sugar Intake

Out of the 22 countries of the EMR, thirteen (59%) had estimates pertinent to TS intake, while only four (18%) had estimates of AS or FS intake. Sugar intake assessments were not identified in the following countries: Djibouti, Iraq, Kuwait, Oman, Qatar, Somalia, Syria and Yemen (Tables S3–S5).

Total Sugar intakes: Some of the studies had estimated TS intakes at the national level for certain age groups (such as adults, children or adolescents), or for the entire population (i.e., per capita), while others had produced estimates pertinent to specific regions within countries [36,37,38,39,40,41,42,43,44,45,46,47,48,49,50,51,52,53,54,55,56,57,58,59,60,61,62]. Table S3 presents data pertinent to TS intakes in countries of the region, whether these intakes were reported as g/day or % EI. Except for Iran, Jordan and Pakistan where TS intake assessment was based on a household budget survey, all the other countries had assessed TS intakes based on dietary assessment approaches such as 24-h recalls, diet records, food diaries, or food frequency questionnaires (Table S3)

**Figure 2:**
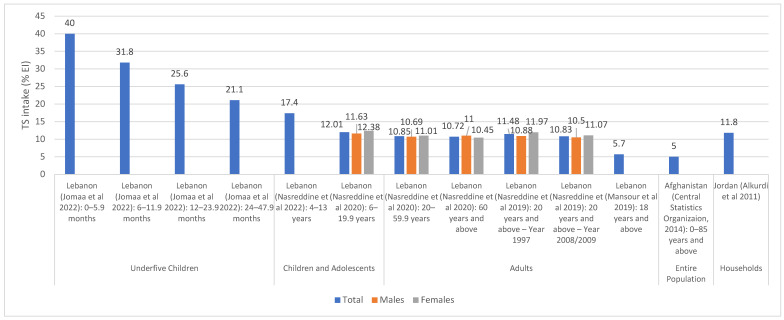
TS intake estimates (% EI) based on national studies in countries of the EMR. Abbreviations: EI: energy intake; EMR: Eastern Mediterranean Region; TS: total sugars. References: For underfive children: Lebanon (dietary assessment): Jomaa et al., 2022 [57]. For children and adolescents: Lebanon (dietary assessment): Nasreddine et al., 2022 [37]; Lebanon (dietary assessment): Nasreddine et al., 2020 [39]. For adults: Lebanon (dietary assessment): Nasreddine et al., 2020 [39]; Lebanon (dietary assessment): Nasreddine et al., 2019 [41]; Lebanon (dietary assessment): Mansour et al., 2019 [42]. For entire population: Afghanistan (consumption data): Central Statistics Organization 2014 [44]. For households: Jordan (Households Income and Expenditure Survey): Alkurdi et al., 2011 [46].

**Figure 3:**
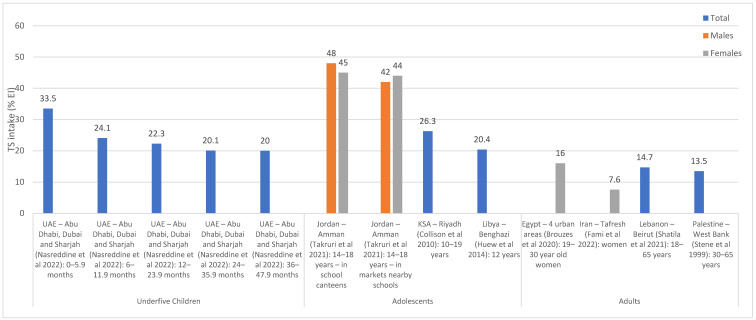
TS intake estimates (% EI) based on regional studies in countries of the EMR. Abbreviations: EI: energy intake; EMR: Eastern Mediterranean Region; KSA: Kingdom of Saudi Arabia; TS: total sugars; UAE: United Arab Emirates. References: For underfive children: UAE (dietary assessment): Nasreddine et al., 2022 [59]. For adolescents: Jordan (dietary assessment): Takruri et al., 2021 [60]; KSA (dietary assessment): Collison et al., 2010 [51]; Libya (dietary assessment): Huew et al., 2014 [52]. For adults: Egypt (dietary assessment): Brouzes et al., 2020 [53]; Iran (dietary assessment): Fami et al., 2002 [54]; Lebanon (dietary assessment): Shatila et al., 2021 [61]; Palestine (dietary assessment): Stene et al., 1999 [55].

Figure 2 and Figure 3 illustrate TS intakes as % EI, based on national and regional studies (i.e., specific regions within a country), respectively. As shown in Figure 2, national surveys conducted in Lebanon (and which were based on dietary assessment approaches) showed that TS intake ranged between 17.4% EI and 40% EI among underfive children, the highest being in 0–6 month old children [57]. In Lebanese children and adolescents, the intake of TS was estimated at 12% EI [39], while in adults, it ranged between 10.5 and 12% EI [39,41]. The study in Afghanistan reported a per capita estimate of 5% EI based on a 7-day food consumption recall [44], while the household budget survey conducted in Jordan reported an estimate of 11.8% EI [46] (Figure 2). As for regional studies (Figure 3), TS intake levels among adults were estimated at 13.5% EI and 14.7% EI in Palestine (West Bank) and Lebanon (Beirut) [55,61], respectively, and rural women in Iran (7.6%) [54], while being slightly higher among urban women in Egypt (16% EI) [53] (Figure 3). Among the few countries that have evaluated TS intake among adolescents, Jordan (Amman) reported the highest levels (42–48% EI) [60], while Libya (Benghazi) reported the lowest (20.4% EI) [52]. TS intakes were also estimated among underfive children in the United Arab Emirates (UAE) (the Emirates of Abu Dhabi, Dubai and Sharjah), ranging between 20% and 33.5%, the highest estimate being observed in 0–6 months old infants [59].

**Figure 4:**
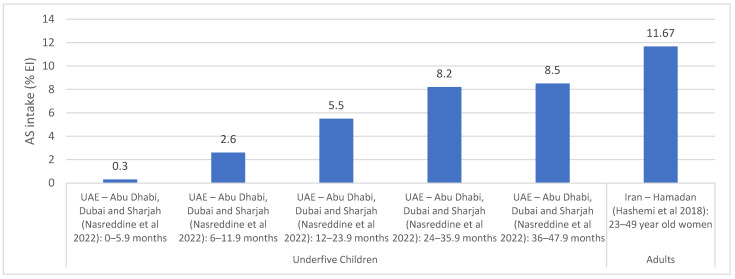
AS intake estimates (% EI) based on regional studies in countries of the EMR. Abbreviations: AS: added sugars; EI: energy intake; EMR: Eastern Mediterranean Region; UAE: United Arab Emirates. References: For underfive children: UAE (dietary assessment): Nasreddine et al., 2022 [59]. For adults: Iran (dietary assessment): Hashemi et al., 2018 [64].

Added sugar and free sugar intakes: AS intakes were assessed based on dietary assessment approaches including 24-h recalls, food diaries, and food frequency questionnaires (Table S4). National studies were only identified in Lebanon with AS intake being estimated at 11.2% EI among 4–13 year old children and adolescents [37]. Other countries have provided estimates based on studies conducted within specific regions [37,38,53,59,63,64], as shown in Figure 4. The UAE (Abu Dhabi, Dubai and Sharjah) reported an average intake ranging between 0.3 and 8.5% among under 4 years old, with the intake increasing with age [59]. In Iran (Hamadan), an intake of 11.7% EI was noted among adult women [64].

**Figure 5:**
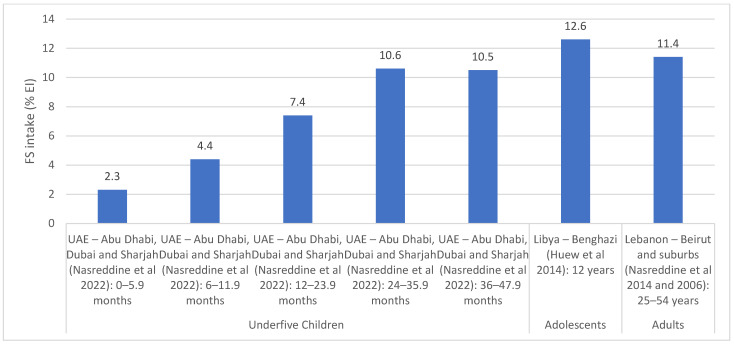
FS intake estimates (% EI) based on regional studies in countries of the EMR. Abbreviations: EI: energy intake; EMR: Eastern Mediterranean Region; FS: free sugars; UAE: United Arab Emirates. References: For underfive children: UAE (dietary assessment): Nasreddine et al., 2022 [59]. For adolescents: Libya (dietary assessment): Huew et al., 2014 [52]. For adults: Lebanon (dietary assessment): Nasreddine et al., 2014 and Nasreddine et al., 2006 [66,67].

Similar to AS, Lebanon was the only country with national estimates for FS intakes, while Libya, Tunisia and the UAE had estimates from specific regions [38,40,52,59,65,66,67,68]. National surveys conducted in 2012 and 2014 in Lebanon showed that the intake of FS ranged between 6.3 and 11.9% EI among underfive children and between 12.6 and 12.9% EI among children and adolescents aged 6–18 years [40] (Table S5). The main contributors of FS were sugar-sweetened beverages (SSBs) (14.9–29.3% of FS intake), biscuits and chocolates (11–12.7%), breads and pies (10.7–12.4%) and sweetened dairy products (9.8%) among under five children, while the main contributors among older children and adolescents were SSBs (13.8–43.5%), biscuits, wafers and chocolates (10.6–14.7%), and syrups, jams and honey (9–9.3%) [38,40]. The regional studies that were conducted in countries of the region are shown in Figure 5. Among adults, FS intake was estimated at 11.4% EI in Beirut, Lebanon [66,67], while in Libya (Benghazi), an intake of 12.6% EI was reported among 12 year old adolescents [52]. A study by Aounallah-Skhiri et al. conducted in 3 regions of Tunisia reported that among adolescents aged 15–19 years (*n* = 1019), FS intake (26.8 g/1000 kcal) exceeded the recommended level (10% EI) [65,69]. The study also showed that sugar and confectionary were among the main constituents of the diet among Tunisian adolescents [65]. Among underfive children, average FS intakes were estimated to range between 2.3 and 10.6% EI among underfive children in the UAE (Abu Dhabi, Dubai and Sharjah) [59], with the intakes increasing with age.

### 3.3. Compliance/Adherence to Sugar Recommendations

Among the few national studies in the EMR, studies conducted among children and adolescents in Lebanon showed that 24.8–54.2% of underfive children and 58.1–62.2% of 6–18 year olds exceeded the WHO FS upper limit (10% EI) [7,38,40]. In the UAE, a regional study on children under the age of 4 years (Abu Dhabi, Dubai and Sharjah) showed that 28% of children aged 12–23.9 months exceeded the upper limit for FS, while 54% and 52% of those aged 24–35.9 months and 36–47.9 months, respectively, exceeded the upper limit for FS [59,70]. A study conducted in KSA (*n* = 424) showed that only 0.9% of children aged 6–12 years consumed FS within the WHO conditional recommendation of <5% EI, whereas 10.6% consumed FS within the recommendation of <10% EI [7,71]. Based on the Tehran Lipid and Glucose Study (*n* = 2510 adults aged 19–70 years), the majority of adults in Tehran, Iran (82.2% of males and 89.8% of females) were found to be adherent to the WHO/Food and Agriculture Organization (FAO) recommendations for FS (i.e., <10% of EI) [69,72].

For AS, national studies conducted in Lebanon showed that only 29.1% of Lebanese children and adolescents were compliant with the AS recommendations set by the American Heart Association (no more than 25 g per day) [37,73] (Table S4). In Sudan, and based on the WHO STEPS survey among adults aged 18 to 69 years, the average weekly intake of AS was estimated at 6.3 teaspoons, which was reported to be within the WHO recommended daily intake of 50 g i.e., 12 level teaspoons [43,74].

### 3.4. Assessment of Sugar Levels in Food and Sugar-Related KAB

Few countries in the EMR (6/22 countries; 27%), including Iran, Jordan, Lebanon, Oman, Qatar and Tunisia, have evaluated sugar levels in local foods and commodities (Table S6) [75,76,77,78,79,80,81,82]. While the majority of available sugar content data were based on the chemical analysis of food products [75,77,78,79,82], few were derived from software analysis (such as ESHA and NutriComp) [76] or nutrition information labels [80]. High levels of sugar were reported in bakery products, ready-to-eat cereals, chocolate and biscuits [80,82]. The lowest levels of sugar (<10 g per 100 g of food) were found in traditional dishes, mayonnaise and salad dressings [75,76,77,78]. Moreover, Iran was the only country that showed decreasing sugar levels in food products (such as salad dressings) between 2017 and 2019 [75].

As for data on KAB, this information was available in 10 EMR countries (45%), including Bahrain, Egypt, Iran, Jordan, KSA, Kuwait, Lebanon, Morocco, Oman and Pakistan. The majority of KAB surveys included questions related to (1) knowledge of sugar food sources, sugar content in food products, and familiarity with adverse weight/health effects of high sugar intakes; (2) consumer’s attitude such as importance of restricting/limiting the intake of sugar, making healthier choices and checking information related to sugar; and (3) consumers’ behavior such as consuming high sugar food products, reducing the amount of AS, substituting high-sugar options with healthier options and reading labels (Table S7) [72,83,84,85,86,87,88,89,90,91,92,93,94,95,96,97,98,99,100,101,102,103,104,105,106,107,108,109,110,111,112,113,114,115,116]. Poor knowledge related to sugar food sources and content was reported from more than 65% of school-aged children in Jazan, KSA [98]. In addition, 77.5% of male schoolchildren in Riyadh, KSA [100], 61% of college students in Muscat, Oman [107] and 85% of adults in Rawalpindi cantonment in Pakistan [111], were unaware of the adverse health effects of high sugar consumption. A national study in Lebanon showed that less than 50% of adolescents and adults did not know what a “sugar-free” claim indicates [116]. As for attitude, more than 80% of schoolchildren in Jazan, KSA preferred high sugar foods and beverages, as compared to healthier options [98]. Among the studies that assessed consumer behavior, a regional study in KSA showed that less than 50% of mothers try to limit the purchase of foods that are high in FS [91], and several studies reported the consumption of high sugar foods and beverages among various age groups. For example, 87.5% of children aged 3–5 years in Zaghouan, Morocco had diets high in sugar [106]; more than 80% of children in Beirut, Lebanon added sugar to their beverages [104]; more than 90% of schoolchildren in Jazan, KSA frequently consumed soft drinks, sweets, milk with sugar and chocolate [98]; 79–83.4% of university students in Karachi, Pakistan consumed cakes, biscuits and soft drinks on a daily basis [113]; and more than 80% of households reported a daily consumption of sugar in Iran [87,88]. In a regional study in KSA, it was reported that only 1.7% of mothers were successful in limiting/controlling their child’s FS intake [91]. Moreover, while 32.6% of female university students in KSA were making an effort to reduce AS intakes [93], only 10.9% of elderly in Nizwa, Oman were restricting sugar in their diet [108].

### 3.5. Countries with National Sugar Reduction Initiatives

Table 1 and Table 2 show the national sugar reduction initiatives that were identified in all of the countries of the EMR, except for Syria (21 countries; 95%).

### 3.6. Leadership and Strategic Approach

National strategies or action plans that express a commitment to reduce sugar in the population were identified in 4 EMR countries; Jordan, KSA, Morocco and Oman (Table 3). All of the identified strategies were led by the government and were part of broader strategies or action plans targeting NCD or healthy diets and lifestyle. Jordan and KSA have specified, in their strategies, the reduction of FS and monosaccharides, respectively [220,221,222].

### 3.7. Implementation Strategies

#### 3.7.1. Taxation, Elimination of Subsidies and Regulation of Marketing

The majority of the EMR countries (19/21 countries; 90%), with the exception of Libya, Somalia and Syria are implementing or planning strategies related to taxation, elimination of sugar subsidies or the regulation of marketing of high-sugar products, with varying degrees in implementation and policy scope (Table 1). Marketing regulation was the least common implementation strategy (10/21 countries; 48%), followed by taxation (13/21 countries; 62%) and then subsidies’ elimination (14/21 countries; 67%).

Taxation is mandatory in 4 countries of the region (Bahrain, KSA, Oman, and Qatar) (Table 1). The first taxation initiatives that were initiated in 2017 in KSA, Qatar, Tunisia and the UAE, have targeted SSBs. KSA and UAE have also extended taxation to include energy drinks and other sweetened food products [118,120,121,132,133,134,135,136,156,158,159,160,161,162]. In addition, Morocco, Oman, Pakistan and Qatar are planning to extend the SSB tax to other sugar rich food products [145,146,147,148,149].

While Iran is implementing a taxation of 10% on local soft drinks and 15% on imported ones (planned to reach 20% on SSBs) [120], Kuwait is implementing a tax of 50% on carbonated beverages and 100% on energy drinks [137]. Oman is already implementing a taxation of 100% on energy drinks and 50% on soft drinks since 2020 [150,151]. Morocco implemented a progressive taxation on sugary drinks in proportion to the quantity of AS (threshold of 5 g/100 mL) [144]. In Afghanistan, there has been a proposal to tax sugary drinks since 2015, but this has not been adopted or implemented yet [117,118]. Similarly, in 2017, Egypt has developed a taxation strategy targeting SSBs; however there is no evidence of its implementation yet [125] (Table 1).

Marketing regulation is mandatory in Jordan and Palestine only (the latter being a planned strategy). While the majority of countries implementing marketing strategies focused on sugar in general (Jordan, Lebanon, Morocco, Palestine, Qatar and UAE) [121,130,131,139,143,155,156,159], other countries have targeted FS (Bahrain) [119], AS (Iran) [127,128], monosaccharides (Iraq) [129] or specifically energy drinks (Pakistan) (Table 1).

Of the 14 countries that have considered subsidies elimination, Oman was the only country that has planned, but not yet adopted, a gradual shift in subsidization from sugars and unhealthy fats to healthy foods instead (to reach 100% by 2025) [152]. Bahrain, is planning to eliminate sugar subsidies [122], while Qatar and the UAE have planned the elimination of any subsidies for sugar-rich food items [156,161,162]. Djibouti, Egypt, Iran, Jordan, Lebanon, Morocco, Pakistan, Sudan, Tunisia and Yemen are eliminating food subsidies for sugar used in industries [123,124,126,138,153,164] (Table 1).

#### 3.7.2. Food Product Reformulation, Consumer Education, Labelling and Interventions in Specific Settings

The majority of the EMR countries (18/21 countries; 86%), with the exception of Djibouti, Egypt, Syria and Yemen are implementing or planning strategies related to food product reformulation, consumer education, labeling or interventions in specific settings. The most common intervention was consumer education (15/21 countries; 71%), followed by interventions in specific settings (13/21 countries; 62%), product reformulation (12/21 countries; 57%) and food labeling initiatives (9/21 countries; 43%). Table 2 displays the details of the initiatives implemented/planned in the various countries.

Twelve countries (57%), including Bahrain, Iran, Jordan, KSA, Kuwait, Morocco, Oman, Pakistan, Palestine, Qatar, Tunisia and the UAE, are implementing/planning product reformulation initiatives in the region. Iran and Kuwait were the first countries in the EMR to have developed food product reformulation strategies on drinks, nectars, biscuits and cakes, in 2016 [168,187]. Morocco will be mandating the reduction of AS by 25% in all foods and beverages by 2025 [198,199]. As for Palestine, mandatory technical instructions have been planned since 2017 to reduce sugar in food products [155]. Similarly, Pakistan has planned a mandatory product reformulation intervention targeting all foods and snacks in 2022 (Table 2). In 2016, Oman has developed an initiative aimed at encouraging food manufacturers to produce smaller-sized portions of products that are high in sugars, but it has not been adopted yet [152].

Fifteen countries (71%) have implemented/planned consumer education campaigns. These countries include Afghanistan, Iran, Jordan, KSA, Kuwait, Lebanon, Libya, Morocco, Oman, Pakistan, Palestine, Qatar, Somalia and the UAE; while Sudan has an initiative that has not yet been adopted (Table 2). Although the majority of awareness and educational campaigns were led by governmental entities in countries of the region, the American University of Beirut and the National Center for Disease Control were the main leaders in Lebanon and Libya, respectively. The WHO, FAO and Aljisr Foundation were collaborators in Oman, and similarly the WHO, FAO, a non-governmental organization (NGO), Pakistan National Heart Association and Diabetic Association of Pakistan were collaborators in Pakistan alongside the government. Libya and Palestine had consumer education initiatives related to AS, while Somalia had initiatives that were specific to FS (Table 2).

Nine countries (43%), including Iran, KSA, Kuwait, Lebanon, Morocco, Oman, Pakistan, Tunisia and the UAE, were found to have implemented/planned food labeling initiatives specific to sugar. Mandatory initiatives were found in Iran, Kuwait, Lebanon, Morocco, Oman, Pakistan and the UAE (7/9 countries; 78%), with the traffic light labeling scheme being implemented in Iran (mandatory) [121,170,171,172,173,174], KSA (voluntary) [182,186] and the UAE (to become mandatory in 2022) [173,217], while it awaits approval in Kuwait (Table 2).

Thirteen countries (62%) are implementing/planning sugar reduction interventions in specific settings. These countries include Bahrain, Iran, Iraq, Jordan, KSA, Kuwait, Lebanon, Morocco, Oman, Pakistan, Palestine, Qatar and the UAE. While all countries have interventions in schools and/or educational institutions, Jordan, KSA, Qatar and the UAE have implemented interventions in other settings. For instance, in Jordan, the Ministry of Health in collaboration with the army sector, has developed an intervention to reduce sugar in menus served to workers and patients in hospitals [177]. As part of the Food & Beverage Guidelines, and the educational sessions in schools and workplaces in Qatar, voluntary guidelines are being implemented in hospitals and the workplace. In the UAE, interventions targeting prisons, juvenile centers, police and military schools, army forces, geriatric home cares and their canteen, as well as health care facilities were developed [161,219]. In KSA, several initiatives have been implemented in various governmental settings (hospitals, universities, military, public and private work environment) and included replacing high-sugar items with healthier options and placing sugar-related claims on sugar packets [182,185] (Table 2).

### 3.8. Monitoring and Evaluation

There are few countries that have conducted monitoring and evaluation activities. In Iran, food items available in primary schools’ canteens in Tehran were evaluated [176], and compliance with the guidelines was assessed by comparing nutrient content of food items (TS, AS and non-sugar sweeteners) with the WHO EMR nutrient profile model [226] and Iran Healthy School Canteen Guideline [175]. The findings showed that 9.2% of chocolates, sugar confectionery and desserts that were available in the school canteens, as well as 15.7% of beverages and 45.2% of cakes, sweet biscuits and pastries, are not permitted based on the WHO EMR model [176,226]. Similarly, 13.7% of chocolates, sugar confectionery and desserts found in the canteens, 19.7% of beverages and 28.8% of cakes, sweet biscuits and pastries are not permitted based on the national guideline [175,176]. Also in Iran, 239 food stores around 64 primary schools in Tehran, were visited in 2018–2019 [227], and the nutrition information on the labels of packaged foods available in these stores were examined. It was found that 89.6% of chocolates, sugar confectionery and desserts, 27.8% of ice cream, 50% of popsicles, 60% of breakfast cereals and 45.9% of cakes, sweet biscuits and wafer had high levels of sugar (classified as greater than 22.5 g per 100 g or greater than 11.25 g per 100 mL or greater than 27 g per serving) [227].

In Jordan, food labels of 663 packaged products in the market were screened for AS, based on the US Food and Drug Administration (FDA) regulations of 2016 and the Jordanian FDA (JFDA) regulations. Although the examined products were compliant with the JFDA regulations, they were not fully compliant with the US FDA standards [228]. A study on 76 public high schools, specifically for boys, in Riyadh, showed that the majority (93–99%) of schools were still offering cakes, muffins, confectionaries, biscuits and cookies [229]. As for Kuwait, and following the implementation of the product reformulation initiative in 2016 to reduce AS content in nectars, fruit juices and SSBs [187], a monitoring and evaluation assessment was conducted in 2017–2018. The findings have shown that the reduction of sugar in nectars ranged between 5–28%, while that of drinks ranged between 2–10% [187].

### 3.9. Impact Assessment

The impact of some taxations schemes was examined. Impact assessment of the sweetened beverage tax was performed in Iran, using data from Iran’s Households Income and Expenditure Survey (HIES) (from 1990 to 2021) [230]. The results showed that the taxation scheme produced a 144.23, 450.04 and 977.94 Kcal monthly decrease in EI per Adult Male Equivalent in all households, low-income households and high-income households, respectively [230].

In KSA, the impact of SSBs taxation was assessed. A cross-sectional study on schoolchildren aged 12–14 years, in Dammam-Khobar-Dhahran cities, showed that there was a decrease in energy drinks consumption (46.1% before tax implementation vs. 38.4% after tax implementation), while the consumption of soft drinks increased (92.5% before tax implementation vs. 94.6% after tax implementation) [136]. However, among adults aged 18–45 years in Medina city, soft drink consumption was shown to decrease by 19% after tax implementation [231]. In terms of annual purchases, the 2004–2018 Euromonitor annual data showed that the annual purchases (volume per capita) of soda and energy drinks were reduced by 41% and 58%, respectively in 2018 (post tax implementation) as compared to 2016 (prior to tax implementations) [232]. Another study measured the impact of sin taxes over the years in KSA, and showed similar findings whereby sales volumes of soft drinks decreased by 57.64% from 2010 to 2017, the year during which sin taxes were implemented [233].

## 4. Discussion

This systematic review reports on sugar reduction initiatives in countries of the EMR, a region that harbors a high burden of obesity, type 2 diabetes and cardiovascular morbidity [1]. It showed that, out of the 22 countries in the EMR, 13 had estimates of TS intakes, while only 4 reported on the intakes of AS or FS. In addition, 21 countries (95%) were found to implement at least one type of sugar reduction initiatives, the most common of which was consumer education (71% out of the countries that have initiatives), while the least common was food labeling initiatives (43% out of the countries that have initiatives).

Although the majority of countries have reported on TS intakes, these intake estimates cannot be compared to health-related reference values given that TS encompasses all sources of sugar, including the intrinsic sugar of whole fruits as well as sugar present in milk and dairy products [5]. This explains the high level of TS observed among infants and young children in Lebanon and the UAE, whereby TS intakes were the highest in infants (31.8–40% EI in Lebanese infants under 1 year [57]; 24.1–33.5% EI in the UAE in the same age group [59]), reflecting their high consumption of milk in this stage of the lifecycle. Although scarce, available data in the region show that AS and FS intakes remain high, exceeding the upper limit of 10% EI in various age groups. For instance, average AS intake was estimated at 11.7% EI among adult women in Iran (Hamadan) [64], while average FS intake was estimated at 11.4% EI among Lebanese adults [66,67]. Data among children and adolescents is particularly alarming. In school-age children, it was reported that 58.1–62.2% of 6–18 year old Lebanese children exceed the WHO FS upper limit (10% EI) [7,38,40], and in KSA, only 10.6% of children aged 6–12 years consumed FS within the recommendation of <10% EI [7,71]. Studies conducted among underfive children also highlight high intakes of sugar in this age group. For instance, 24.8–54.2% of underfive children in Lebanon [7,38,40] and 28–54% in the UAE [59,70] were found to exceed the upper limit of FS (10% EI).

The present review shows that multicomponent sugar reduction approaches have been implemented in several countries of the region. This is a positive finding since an integrated policy approach may further foster the impact and effectiveness of sugar reduction strategies [35,234,235]. The WHO EMR policy statement for lowering sugar intake [26], has in fact included recommendations for multifaceted interventions for the reduction of sugar intake, including financial policies, food reformulation, food labelling, consumer education, marketing regulations and interventions in specific settings. The most common sugar reduction approach in the EMR was consumer education, which was adopted by 15 countries in the region (71%), with the aim of raising awareness about sugar, its main dietary sources and its potential adverse health effects. For example, the reduction of sugar intake has been included in country-specific food-based dietary guidelines in Afghanistan, Iran, Jordan, KSA, Lebanon, Libya, Oman, Pakistan, Palestine, Qatar and the UAE. Such guidelines and their dissemination to the public has been reported as a practical and effective approach in ameliorating dietary knowledge and attitudes among consumers [236,237]. Some studies in the region had in fact assessed consumers’ knowledge and attitudes towards sugar. These studies have shown that consumers have poor or little knowledge related to the sources of sugar or their content in foods [98], as well as the potential health effects of high sugar intake [100,107,111]. A negative attitude towards sugar reduction or the consumption of healthier low-sugar foods had been also reported from countries of the region [98]. In addition, from the few available studies on practices, a study in KSA showed that less than half of mothers try to limit the purchase of foods that are high in FS [91], and that only 1.7% of mothers were successful in limiting their child’s FS intake [91]. Similarly, it has been reported that high proportions of school-aged children or university students add sugar to their beverages [104] and consume SSBs and sweets on a daily basis [98,113]. It is recommended that countries of the region refer to findings stemming from the available studies on population, knowledge, attitude and practice to further tailor their consumer education initiatives and address culture-specific knowledge gaps and barriers against sugar reduction.

Fiscal policies have been implemented in various countries of the region, including sugar subsidies’ elimination (14 countries; 67%) and taxation (13 countries each; 62%). These estimates highlight a significant progress, compared to earlier reports from the region. In 2016, the first Global Nutrition Policy Review reported that only 24% of countries in the EMR were implementing fiscal policies [238], while our findings show that this proportion has increased to 77% of the 22 countries of the region. The EMR as a region has had a sugar subsidizing policy as part of a strategy to support the poorer sections of the community [26]. However, the importance of eliminating sugar subsidies has recently gained momentum in the region, basically driven by the WHO EMR. For instance, the EMR policy statement on sugar reduction clearly states that countries ought to “review national food subsidies and progressively reduce and finally remove them as a policy mechanism for improving the health of the poor and access to health services” [26]. In Oman, and as part of the National plan for the prevention and control of chronic NCDs 2016–2025, there is a plan to gradually shift subsidization from sugars and unhealthy fats to healthy foods instead, to reach 100% by 2025 [152].

The implementation of taxation policies in the region was found to vary between countries with respect to the tax rate (as percentage of price), the included beverages, and whether taxation was extended to sugar-rich foods. Taxation policies in countries of the region have mainly focused on SSBs (13 countries). However other countries have included other beverages such as energy drinks (7 countries) and juices (3 countries), with some countries also implementing/planning to extend the taxation to sugar-containing foods (2 countries are implementing and 4 are planning). While the majority of countries have implemented a taxation of 50% on SSBs (such as Bahrain, KSA, Kuwait, Morocco, Oman, Qatar and the UAE), Iran is planning to reach 20%. In addition, several countries (Bahrain, Kuwait, Oman, Qatar and the UAE) have implemented a taxation rate of 100% on energy drinks, while KSA has adopted a taxation rate of 120%. Available evidence from various parts of the world suggests that SSB taxation leads to a reduction in the sales of taxed beverages, although studies examining the influence of SSB taxation on consumption have produced mixed results [34,35]. Taxation effects on sales and intake may in fact depend on several country-specific factors such as taxation rate, baseline SSB intake, type of beverage taxed, as well as population demographics [34].

Thirteen countries (62%) in the region have implemented sugar-reduction interventions in specific settings (mandatory or voluntary). Together with other initiatives and policies, these interventions could send a clear and coherent message to the public and industry, and address exposure to high sugar foods and beverages in the many places where people learn, work, and play [34]. While the most common setting for these interventions were schools in the EMR, some countries have implemented sugar-reduction initiatives in the workplace and in hospitals. The nature of these interventions varied between countries but mainly aimed at limiting the availability of SSBs/foods in these settings. Such interventions can in fact decrease the target population’s exposure to sweetened beverages/foods while making healthier beverages more accessible [34]. Research investigating the impacts of availability restriction on consumption is limited, but available evidence is promising [34]. A study investigating the impact of a workplace SSB ban at a California hospital showed that employees who were previously regular SSB consumers had reduced their daily intake by approximately half and had witnessed significant reductions in their waist circumference measurements [239].

Product reformulation has been implemented by 12 countries in the region (57%), mainly through setting targets/standards for sugar, either through mandatory regulation or co-regulation with the food-industry. Food reformulation is in fact one of the interventions recommended by the WHO to improve the nutritional quality of the food supply and hence the population’s diet [240]. Available studies suggest that policies targeting the reformulation of foods are cost-effective [35,241], and that these policies may have greater health impacts than those focusing on changing consumer behavior [242,243]. At the international level, there are few examples of policies setting targets/standards for AS or FS in packaged foods. Since 2008 in France, 37 food manufacturers and retailers have signed the government’s Charters of Voluntary Engagement to reduce sugar in their products [35]. It was then estimated that close to 13,000 tonnes of sugar were removed from the French food market between 2008 and 2010 [244]. In the UK, the health impact of three policy scenarios on health (obesity, diabetes, dental carries) were assessed, including product reformulation, increasing product price, or changing the market share of high-sugar, mid-sugar, and low-sugar drinks [245]. Out of the three modelled scenarios, the best was found to be reformulation [245].

The regulation on the marketing of high-sugar foods and beverages was found to be implemented/planned in 10 countries of the region (48%). The marketing of high sugar foods and beverages is becoming increasingly aggressive in the EMR as the Region represents a suitable marketing opportunity due to the limited regulatory restrictions [26]. Carefully constructed counter-marketing campaigns, which have led to efficient reductions in SSB sales in the US and Australia [246,247], were described as effective tools that could complement any sugar reduction policy. The EMR policy statement on sugar reduction highlighted the need for special measures to address the unopposed marketing of high sugar foods and beverages and to regulate marketing of unhealthy food and drinks to children through the use of the WHO regional nutrient profile model [26,226].

Food labeling initiatives (9 countries; 43%) were found to be the least implemented sugar reduction initiative in the region. Front of pack labeling, mainly the traffic light, has been implemented mandatorily in Iran, and on a voluntary basis in KSA, Kuwait and the UAE (with a plan to become mandatory in the latter as of 2022). Front-of pack labels provide simple and easy-to-understand information that help the consumer in making healthier food choices [34], while also incentivizing the food industry to reformulate their products [34]. Front-of-pack labels are expected to have a larger impact on consumer’s purchasing behavior and food choice, as compared to the numeric information found in the Nutrition Facts Panel on the side or back of food packages. The latter approach (numeric nutrient facts panel) was found to be implemented by 5 countries in the region, thus highlighting the need for more efforts on the promotion and implementation of front of pack labeling in the EMR. It is also important for labelling schemes implemented in the region to focus on AS instead of TS per se. A recent meta-analysis found that food labelling initiatives (including back of pack and menu labelling) that focus on TS only were not associated with any significantly reduction in sugar content in foods [248].

This systematic review showed that some countries have included a legislative component within their sugar reduction strategies, instead of solely implementing voluntary initiatives. It was previously shown that mandatory or legislative approaches tend to be more effective, leading to more significant reductions in sugar intakes within the population, compared to voluntary strategies [35]. A recent review has indicated that, although all policy approaches may result in reductions in the levels of sugar in foods and subsequent intakes, stronger policies (i.e., mandatory ones) will carry more pronounced impacts than voluntary food reformulation or labeling initiatives [35].

The implementation of clear monitoring activities is crucial to show program effectiveness, and to spur greater impact on sugar reduction [249,250]. In the EMR, few countries have conducted monitoring activities related to their sugar reduction initiatives. These included Iran, Jordan, KSA and Kuwait, although none had clearly established mechanisms for the monitoring of sugar reduction initiatives/programs. The few countries that have conducted monitoring activities have reported poor compliance. For instance, the majority of schools (93–99%) in Kuwait are still offering cakes, muffins, confectionaries, biscuits and cookies [229], and a considerable proportion of foods and beverages (9.2–45.2%) available in schools in Iran were described as “not permitted” based on the Iran Healthy School Canteen guidelines [175,176,226].

Data on the impact of sugar reduction initiatives is limited in the region. Except for Iran and KSA, there were no studies or reports examining the impact of sugar reduction policies in the other countries of the EMR. In Iran, the impact of the sweetened beverage tax was assessed, and the results showed that the taxation scheme produced a 144.23 Kcal monthly decrease in EI per Adult Male Equivalent [230]. In KSA, the impact of SSBs taxation was assessed. In terms of annual purchases: the Euromonitor annual data showed that the annual purchases of soda and energy drinks were reduced by 41% and 58%, respectively, post tax [232]. Although there was a decrease in the consumption of energy drinks among school-aged children [136], and soft drinks among adults [231], the intake of soft drinks seems to have increased among school-aged children (92.5% before tax implementation vs. 94.6% after tax implementation) [136]. The scarcity of impact data in the region, may be partially due to the fact that many sugar reduction initiatives are relatively recent and there has been insufficient time to allow for the assessment of impact. There is a crucial need for well-constructed impact evaluations in countries of the region. In addition, since the regular evaluation of changes in population sugar intake and sugar levels in foods may be costly and complex, the inclusion of process evaluations that assesses policy implementation and its progress, collects data on process indicators, and identifies existing facilitators and barriers to the implementation is also crucial in providing real-time information, and highlighting specific areas for improvement [251].

This review has a number of strengths and limitations. It is the first systematic review of existing sugar reduction initiatives and policies in countries of the EMR. The review relied on a systematic search of databases and grey literature, but also sought additional input from focal points or program leaders in the various countries of the EMR, to confirm and obtain supplementary country-specific data. Although we could not identify all country contacts and there were some non-respondents, the triangulation of information from various sources allowed us to identify and characterize the existing initiatives and implementation strategies, and present the information in a relatively standardized approach. Through this multifaceted methodology, it is unlikely that any major sugar reduction initiatives were missed, although this possibility cannot be totally discounted. While one of the important strengths of the review is the fact that it included an inclusive search of the grey literature, encompassing governmental reports and questionnaires completed by country program leaders, a possible limitation of this approach is the fact that the methodological rigor within some of these reports/questionnaires was not ascertained. More specifically, the robustness of the studies’ designs, their methodology and the quality of the data used for the evaluation of sugar intake and sugar levels in foods were not examined and hence data should be interpreted with caution. It is also important to note that studies that have reported on the intakes of AS and FS were very scarce and that dietary estimation of AS/FS sugar intake levels is inherently limited by the scarcity of up-to date, culture-specific food composition databases, and by the poor consumer knowledge of the potential food sources of AS/FS.

## 5. Conclusions

This systematic review showed that, despite the scarcity of data, the intakes of AS and FS remain high in countries of the region, exceeding the upper limits set by the WHO in all age groups, especially among children and adolescents. Except for Syria, all countries were found to implement at least one type of sugar reduction initiatives, the most common being consumer education, followed by fiscal policies (sugar subsidies’ elimination and taxation), interventions in public institution settings, product reformulation, marketing regulations and finally food labeling initiatives. A positive finding of this review is that several countries were found to implement multicomponent interventions for sugar reduction, which, as described in the literature, are expected to exert a larger impact compared to single policy initiatives [34]. However, data on the monitoring and impact assessment of the implemented sugar-reduction initiatives was very limited in the region. Further action is needed to make sure that countries strengthen their regulatory capacities and compliance monitoring of sugar reduction policy actions, and meet the targets sets in their national action plans and strategies.

## Figures and Tables

**Table 1:** Sugar reduction implementation strategies in countries of the EMR: Taxation, subsidies and marketing regulations.

Country	Taxation	Subsidies	Marketing Regulations
Afghanistan	Name of initiative: National Health Policy 2015–2020	-	-
	Year: 2015		
	Leadership: Led by the government (MOPH)		
	Approach and target: Taxation on drinks with AS will be considered (not adopted) [117,118].		
Bahrain	Name of initiative: National Action Plan for control and prevention of Non communicable diseases (2019–2030)	Name of initiative: NCD Joint Programming Mission	Name of initiative: National Action Plan for control and prevention of Non communicable diseases (2019–2030)
	Year: 2019	Year: 2017	Year: 2019
	Leadership: Led by the government (MOH)	Leadership: Led by the government (MOH)	Leadership: Led by the government (MOH)
	Approach and target: Mandatory: Taxation on energy drinks and carbonated beverages [119]; reaching 100% on energy drinks and 50% on SSBs [120,121].	Approach and target: Remove subsidies on locally produced juices (not adopted) [122].	Approach: Policies to reduce the impact of marketing of foods and nonalcoholic beverages high in FS, on children (not adopted) [119].
Djibouti	-	Name of initiative: Sugar subsidies law	-
		Year: NA	
		Leadership: NA	
		Approach: Eliminating food subsidies for sugar used in industries (pastries, candies, chocolate, sweets…) [123,124].	
Egypt	Name of initiative: National Multisectoral Action Plan for Prevention and Control of Noncommunicable Diseases	Name of initiative: Sugar subsidies law	-
	Year: 2017	Year: NA	
	Leadership: Led by the government (MOHAP)	Leadership: NA	
	Approach and target: Taxation on SSBs to reduce sugar consumption (not adopted) [125].	Approach: Eliminating food subsidies for sugar used in industries (pastries, candies, chocolate, sweets…) [123,124].	
Iran	Name of initiative: Sweetened beverage tax	Name of initiative: Sugar subsidies law	Name of initiative: 5th national development plan (2011–2016)
	Year: NA	Year: NA	Year: 2011
	Leadership: NA	Leadership: NA	Leadership: Led by the government
	Approach and target: Tax of 10% on local soft drinks and 15% on imported ones; to reach 20% on SSBs [120].	Approach: Eliminating food subsidies for sugar used in industries (pastries, candies, chocolate, sweets…) [123,126].	Approach: Marketing foods with AS is forbidden. Soft drinks and 19 unhealthy food products (such as edible ice products, toffee and candy) have been banned [127,128].
Iraq	-	-	Name of initiative: National strategy for the prevention and control of non-communicable diseases 2018–2022
			Year: 2018
			Leadership: Led by the government (MOH, Ministry of Justice and the parliament)
			Approach and target: Limiting the marketing of food items that are high in monosaccharides, to children [129].
Jordan	-	Name of initiative: Sugar subsidies law	Name of initiative: National Framework of Action on Obesity Prevention in Jordan 2018–2023
		Year: NA	Year: 2019
		Leadership: NA	Leadership: Led by the government (MOH), in collaboration with Jordan Food and Drug Administration, Jordan Standards and Metrology Organization, Ministry of Education, Amman Municipality, Ministry of Youth, Ministry of Industry and Trade, the Royal Medical Services, Ministry of Planning and the University of Jordan
		Approach: Eliminating food subsidies for sugar used in industries (pastries, candies, chocolate, sweets…) [123,124].	Approach: Mandatory: Restrict marketing of foods high in sugar [121,130,131].
KSA	Name of Initiative: Sin Taxation	-	-
	Year: 2017		
	Leadership: Led by the government (SFDA)		
	Approach and target: Mandatory: Taxation was imposed by the General Authority of Zakat, Tax and Customs (GZAT) as of 2017; Enforcement of a flat tax (in 2019) of 50% on SSBs, including carbonated drinks, juices, and dairy products and 120% on energy drinks. In 2018, an additional 5% taxation was implemented on beverages [120,121,132,133,134,135,136].		
Kuwait	Name of initiative: Taxation	-	-
	Year: 2020		
	Leadership: Led by the GCC Standardization Organization		
	Approach and target: Implementing excise tax on SSBs (50% on carbonated beverages and 100% on energy drinks) [137].		
Lebanon	-	Name of initiative: Sugar subsidies law	Name of initiative: NA
		Year: NA	Year: NA
		Leadership: NA	Leadership: Led by Consumers Lebanon
		Approach: Eliminating food subsidies for sugar used in industries (pastries, candies, chocolate, sweets…) [123,138].	Approach: Advocacy with the Association of Lebanese Artists to refrain from participation in the creation of advertisements that promote foods high in sugar [139].
Morocco	Name of initiative: National Multisectoral Strategy for the Prevention and Control of Non-Communicable Diseases 2019–2029	Name of initiative: Sugar subsidies law	Name of initiative: National Nutrition Program; National Multisectoral Strategy for the Prevention and Control of Non-Communicable Diseases 2019–2029
	Year: 2019	Year: NA	Year: 2019
	Leadership: Led by the government (MOH) and General Directorate of Customs and parliament	Leadership: Led by the parliament	Leadership: Led by the government (MOH)
	Approach and target: Tax on SSBs are 50% [120,140]; the soda tax was adopted by the Moroccan Parliament in the 2019 finance bill [141,142].	Approach: Eliminating food subsidies for sugar used in industries (pastries, candies, chocolate, sweets…) [123,124].	Approach: Regulate the marketing of high-sugar products [140,143].
	Name of initiative: Progressive taxation		
	Year: 2020		
	Leadership: Led by the government (Ministry of Finance) and parliament		
	Approach and target: A progressive taxation on sugary drinks in proportion to the quantity of AS (threshold of 5 g/100 mL) [144].		
	Name of initiative: Excise tax		
	Year: 2022		
	Leadership: Led by the government (MOH) and parliament		
	Approach and target: The sugar tax has been adopted by the moroccan parliament in the 2023 finance bill (11 November 2023). It includes processed products containing sugar such as cookies, industrial pastry, wafers, breakfast cereals, cereal bars, dairy products, drink yogurt, milk desserts, sweetened condensed milk, chocolates, confectionery [145,146,147,148,149].		
Oman	Name of initiative: Oman Ministerial decree No. 112/2019 on determining the value and types of selective goods and the category of tax levied on each	Name of initiative: National plan for the prevention and control of chronic non-communicable diseases 2016–2025	Name of initiative: National plan for the prevention and control of chronic non-communicable diseases 2016–2025
	Year: 2019	Year: 2016	Year: 2016
	Leadership: Led by the government (Ministry of Finance)	Leadership: Led by the government (MOH)	Leadership: Led by the government (MOH)
	Approach and target: Mandatory: Taxation on SSBs (fruit juices and nectars, energy drinks, canned and prepared coffee and tea beverages) and carbonated beverages; 100% for energy drinks and 50% for soft drinks; to be increased to 100% for SSBs [150,151].	Approach and target: Gradually shift subsidization from sugars and unhealthy fats to healthy foods instead–to reach 100% by 2025 (not adopted) [152].	Approach: Prevent marketing of non-healthy food for children by 2025 (not implemented) [152].
	Name of initiative: Policy statement for sugar reduction		
	Year: 2022		
	Leadership: Led by the government (MOH)		
	Approach and target: Voluntary: Meetings with companies have been conducted during the months of July and October 2022; Taxation on high-sugar products (needs to be finalized).		
Pakistan	Name of initiative: National Action Plan for Reduction of Dietary Risks of NCDs for Pakistan	Name of initiative: Sugar subsidies law	Name of initiative: NA
	Year: 2022 (Planned)	Year: NA	Year: NA
	Leadership: Led by the government (Ministry of National Health Services, Regulation and Coordination), in collaboration with the WHO	Leadership: NA	Leadership: Led by the Punjab Food Authority
	Approach and target: Will be mandatory for all foods and snacks (bakery products, breakfast cereals, desserts, puddings and ice cream, SSBs, biscuits and cakes, ultra-processed foods): Approach includes taxation for high sugar products.	Approach: Eliminating food subsidies for sugar used in industries (pastries, candies, chocolate, sweets…) [123,153].	Approach and target: Marketing of energy drinks has been banned in the geographic limits of the province.
	Information provided by the NFP		Information provided by the NFP
	Name of initiative: Saving Youth from Sugar-sweetened Beverages through Education, Media, and Advocacy (SYSTEM)		
	Year: 2020		
	Leadership: Pakistan National Heart Association; in collaboration with MOH, MOF, Federal Board of Revenue and other policy makers		
	Approach and target: Advocate for SSB taxation [154].		
Palestine	Name of initiative: NA	-	Name of initiative: National Health Strategy 2017–2022
	Year: 2022		Year: 2017
	Leadership: Led by the government (Ministry of Finance and Planning)		Leadership: Led by the government (MOH) in cooperation with all health sector stakeholders
	Approach and target: Planned taxation on carbonated beverages, energy drinks, sweetened juices and juice concentrates.		Approach: Planned mandatory technical instructions regulating the marketing of foods containing high sugar content (planned; not implemented yet) [155].
	Information provided by the NFP		
Qatar	Name of initiative: Qatar National Nutrition and Physical Activity Action Plan 2017–2022	Name of initiative: Qatar National Nutrition and Physical Activity Action Plan 2017–2022	Name of initiative: Qatar National Nutrition and Physical Activity Action Plan 2017–2022
	Year: 2017	Year: 2017	Year: 2017
	Leadership: Led by the government (MOPH)	Leadership: Led by the government (MOPH)	Leadership: Led by the government (MOPH)
	Approach and target: Taxation to raise the price of sweetened soft drinks and beverages [156].	Approach and target: Progressive elimination of any subsidies for sugar-rich food items [156].	Approach: Restrict the marketing, advertising and sponsorship across all media (including digital) platforms for all sugar-rich foods and drinks to children and adults (not adopted); actions to limit price promotions on foods high in sugar in supermarkets, catering or street markets [156].
	Name of Initiative: Establishment of General Tax Authority		
	Year: 2018		
	Leadership: Led by the government (MOF)		
	Approach and target: Mandatory: Impose 100% tax on energy drinks and 50% tax on sugary drinks [120,157].		
	Name of initiative: Taxation for high sugar products		
	Year: Planned		
	Leadership: Led by the government		
	Approach and target: Taxation on bakery products, desserts, SSBs, biscuits and cakes.		
	Information provided by the NFP		
Sudan	-	Name of initiative: Sugar subsidies law	-
		Year: NA	
		Leadership: NA	
		Approach: Eliminating food subsidies for sugar used in industries (pastries, candies, chocolate, sweets…) [123,124].	
Tunisia	Name of initiative: NA	Name of initiative: Sugar subsidies law	-
	Year: 2014	Year: NA	
	Leadership: Led by the government (Ministry of Commerce)	Leadership: NA	
	Approach and target: Prices of sugar have been increased first for the food industry and then for the consumer [158].	Approach: Eliminating food subsidies for sugar used in industries (pastries, candies, chocolate, sweets…) [123,126].	
	Name of initiative: National Obesity Control and Prevention		
	Year: 2017		
	Leadership: Led by the government (Ministry of Commerce) with the cooperation of industries		
	Approach and target: Taxation on SSBs [118,120,158].		
UAE	Name of initiative: National Plan to Combat Childhood Obesity United Arab Emirates; National Plan for Prevention and Response For noncommunicable diseases; National Action Plan in Nutrition	Name of initiative: National Action Plan in Nutrition; National Plan for Prevention and Response to NCDs 2017–2021	Name of initiative: National Plan to Combat Childhood Obesity United Arab Emirates
	Year: 2017	Year: 2017	Year: 2017
	Leadership: Led by the government (MOHAP–Department of Health Education and Promotion)	Leadership: Led by the government (MOHAP)	Leadership: Led by the government (MOHAP–Department of Health Education and Promotion)
	Approach and target: Implement taxes on SSBs according to the WHO guidance [159,160,161].	Approach and targets: Government subsidies on high-sugar foods removed [161,162].	Approach: Limit the consumption of foods and beverages high in sugar, to infants and young children through marketing regulations that are in line with the WHO recommendations (not adopted) [159].
	Name of initiative: Cabinet Decision No. 52 of 2019 on Excise Goods, Excise Tax Rates and the Methods of Calculating the Excise Price		
	Year: 2019		
	Leadership: Led by the government (Federal Tax Authority)		
	Approach and targets: 50% on carbonated beverages; 100% on energy drinks; 50% on any product with AS or other sweeteners [120,163].		
Yemen	-	Name of initiative: Sugar subsidies law	-
		Year: NA	
		Leadership: NA	
		Approach: Eliminating food subsidies for sugar used in industries (pastries, candies, chocolate, sweets…) [123,164].	

Abbreviations: AS, added sugars; EMR, Eastern Mediterranean Region; FS, free sugars; GCC, Gulf Cooperation Council; GZAT, General Authority of Zakat, Tax and Customs; KSA, Kingdom of Saudi Arabia; MOF: Ministry of Finance; MOH, Ministry of Health; MOHAP, Ministry of Health and Prevention; MOPH, Ministry of Public Health; NA, not available; NCD, non-communicable disease; NFP, nutrition focal point; SFDA, Saudi Food and Drug Authority; SSB, sugar-sweetened beverages; UAE, United Arab Emirates; WHO, World Health Organization.

**Table 2:** Sugar reduction implementation strategies in countries of the EMR: product reformulation, consumer education, labeling and interventions in specific settings.

Country	Product Reformulation	Consumer Education/Behavior Change	Labeling	Interventions in Specific Settings
Afghanistan	-	Name of initiative: National Health Policy 2015–2020	-	-
		Year: 2015		
		Leadership: Led by the government (MOPH)		
		Approach: Promote health and highlight the risk of over consumption of foods high in sugar (not adopted) [117].		
		Name of initiative: Guideline to reduce sugar intake and avoid sweet carbonated beverages Year: 2016		
		Leadership: Led by the government (MOPH, Ministry of Agriculture, Irrigation and Livestock, and Ministry of Education, in collaboration with the WHO)		
		Approach: Inclusion in FBDG [165].		
Bahrain	Name of initiative: NCD Joint Programming Mission	-	-	Name of initiative: Food canteen list for the academic year 2016–2017
	Year: 2017			Year: 2016
	Leadership: Led by the government (MOH)			Leadership: Led by the government (MOH)
	Approach and target: Work on the law to reduce sugar in locally made juices (not adopted) [122].			Approach: List of allowed and not allowed food items (which include some high sugar items such as fruit/nectar drinks, soft drinks, energy drinks, sweet biscuits, croissants, sweets and candies)
				Setting: Schools [121,166,167].
Iran	Name of initiative: NA	Name of initiative: Guideline on the consumption of sugar, sweet foods and beverages, and soft drinks	Name of the Initiative: Nutritional Traffic Light labeling	Name of initiative: National Guidelines for Healthy School Canteen
	Year: 2016	Year: 2015	Year: 2016	Year: 2014
	Leadership: Supreme Council for Health and Food Security	Leadership: Led by the government (Ministry of Health and Medical Education; with the involvement of the Tehran University of Medical Sciences; Iranian Nutrition Society; and the National Nutrition and Food Technology Research Institute, Tehran, as well as NGOs and the WHO EMRO	Leadership: Led by the government	Leadership: Led by the government (Ministry of Health and Medical Education in collaboration with MOH)
	Approach and target: Reformulate foods to reduce sugar primarily in drinks, by up to 10% [168].	Approach: Inclusion in FBDG [169].	Approach: Started in 2014 as voluntary; became mandatory in 2016; mandatory label that indicates the content of sugar in foods, as follows: Low (Green): less than or equal to 5 g per 100 g or less than or equal to 2.5 g per 100 mL Medium (Yellow): greater than 5 g to less than or equal to 22.5 g per 100 g, or greater than 2.5 g to less than or equal to 11.25 g per 100 mL High (Red): greater than 22.5 g per 100 g; greater than 11.25 g per 100 mL; greater than 27 g per serving [121,170,171,172,173,174].	Approach: Prohibited the marketing of soft drinks and sweet biscuits; all schools should provide healthier food and drink options in school canteens
				Setting: Schools [167,175,176].
	Name of initiative: Iran National Standard	Name of initiative: Educational campaigns for increasing awareness on salt and sugar, total fat and TFA reduction		
	Year: NA	Year: Annually since 2010		
	Leadership: Led by the National Standard Organization	Leadership: Led by the government		
	Approach and target: Reducing the sugar content in biscuit products by 10% and in flavored milk from 8% to 6%.	Approach: Social marketing (e.g., campaigns), TV advertising and events.		
	Information provided by the NFP	Information provided by the NFP		
Iraq	-	-	-	Name of initiative: National strategy for the prevention and control of non-communicable diseases 2018–2022
				Year: 2018
				Leadership: Led by the government (MOH, Ministry of Education, Ministry of Youth and Sports and Ministry of Commerce)
				Approach: Limiting the marketing of food items and beverages that are high in monosaccharides, to children in the school setting
				Setting: Schools [129].
Jordan	Name of initiative: National Framework of Action on Obesity Prevention in Jordan 2018–2023	Name of initiative: Low salt, low sugar, low saturated and trans fat consumption guideline for health care providers for training of trainers (TOT) and pamphlet for consumers	-	Name of initiative: Health requirements for school canteens and foods allowed and prohibited to be sold for the year 2012
	Year: 2019	Year: 2015		Year: 2012
	Leadership: Led by the government (MOH), in collaboration with Jordan Food and Drug Administration, Jordan Standards and Metrology Organization, Ministry of Education, Amman Municipality, Ministry of Youth, Ministry of Industry and Trade, the Royal Medical Services, Ministry of Planning and the University of Jordan Approach and target: Food reformulation regulations to reduce sugar, guided by WHO recommendations [121,131,177].	Leadership: Led by the government (MOH)		Leadership: Led by the government (Ministry of Education)
		Approach: Awareness and education [178].		Approach: Food items have been prohibited from being sold at school canteens; they include soft drinks of all kinds, drinks containing sugar, candies, chocolates, lollipops, ice cream, biscuits of all kinds and prepackaged cakes
				Setting: Schools [167,179].
		Name of initiative: Guideline for low consumption of salt, sugar, saturated fat and trans fat		Name of initiative: NA
		Year: 2020		Year: NA
		Leadership: Led by the government		Leadership: Led by the government (MOH), and the army sector
		Approach: Inclusion in FBDG [180].		Approach: Reduce sugars in menus served to the workers and patients at hospitals
				Setting: Hospitals [177].
KSA	Name of initiative: Healthy Food Strategy, vision 2030	Name of initiative: Guideline for low consumption of sugar	Name of initiative: A guide for healthy food terms and conditions in government subsistence purchase contracts	Name of initiative: A guide for healthy food terms and conditions in government subsistence purchase contracts
	Year: 2018	Year: 2012	Year: 2019	Year: 2019
	Leadership: Led by the government (SFDA)	Leadership: Led by the government (MOH)	Leadership: Led by the government (SFDA)	Leadership: Led by the government (SFDA)
	Approach and target: Mandatory: Reduce the sugar content in food products by spreading awareness among food manufacturers and importers [133].	Approach: Inclusion in the Dietary Guidelines for Saudis-The Healthy Food Palm [181].	Approach: Nutrition labels ought to include TS and AS. Nutrition claims should be as follows: (1) “low in sugar” for products that do not contain more than 5 g of sugars per 100 g of solid food or more than 2.5 g of sugars per 100 mL of liquids; (2) “sugar-free” for products that do not contain more than 0.5 g of sugars per 100 g or per 100 mL; (3) “no added sugars” for products that do not contain any added monosaccharides or disaccharides or any other sweetener; (4) “reduced sugar” for products that contain the same amount of energy or less energy as compared to similar products [120,132,173,182,183]	Approach: Implementing healthy food terms and conditions: (1) select products that contain no AS or products that are low in AS as per the SFDA.FD 2233/2018; (2) replace chocolates with granola, fruits, unsalted nuts; (3) replace carbonated drinks with water, 100% fruit juices, milk low in fat; (4) offer sweets that are low in energy such as low-fat zabadi
				Setting: Governmental settings (hospitals, universities, military) [182].
	Name of Initiative: SFDA.FD 5001: Fresh juices, mixes and beverages, sold at juice stores, restaurants and cafes			Name of initiative: An initiative to promote public health through food in the work environment
	Year: Issued in 2019 and enforced in 2020			Year: 2019
	Leadership: Led by the government (SFDA)			Leadership: General Authority for Food and Medicine
	Approach and target: (1) Regulate the nutrient composition in fresh juices, nectars and fruit drinks and their display on products; (2) Encourage food and beverage establishments (restaurants, hotels, coffee shops, juice shops and supermarkets that sell juices) to withhold adding sugars to fresh juices, nectars and fruit drinks [184].			Approach: In addition to those mentioned by the “Guide for healthy food terms and conditions in government subsistence purchase contracts”, (1) prohibit the provision of carbonated beverages; (2) offer fresh juices rather than artificial juices or nectar; (3) avoid adding sugars to fresh juices; (4) Place the following claim on sugar packets “The WHO recommends a maximum of 50 g of sugar per person per day
				Setting: Public and private work environment [185].
			Name of initiative: SFDA.FD 42 “Traffic light labeling”	Name of initiative: NA
			Year: 2019	Year: NA
			Leadership: Led by the government (SFDA)	Leadership: NA
			Approach: Voluntary for all food categories Low (Green): less than or equal to 5 g per 100 g Medium (Orange): greater than 5 g to less than or equal to 22.5 g per 100 g High (Red): greater than 22.5 g per 100 g; greater than 27 g per serving [182,186].	Approach: Prohibited the marketing of soft drinks and sweet biscuits
				Setting: Schools [167].
Kuwait	Name of initiative: Sugar reduction initiative	Name of initiative: Kuwait Food Based Dietary Guidelines	Name of initiative: Labeling technical regulation (Requirements of Nutritional Labeling GSO 2233/2012)	Name of initiative: The Case for Investment in Prevention and Control of Non-Communicable Diseases in Kuwait
	Year: 2016	Year: 2022	Year: 2012	Year: 2020
	Leadership: Led by the government (Community Nutrition Promotion Sector (CNPS) of Public Authority for Food and Nutrition (PAFN)), on behalf of the Kuwait MOH, in partnership with the WHO and the food industries	Leadership: Led by the government	Leadership: Led by the GCC Standardization Organization	Leadership: Led by the FNA on behalf of the Kuwait MOH, in partnership with the WHO and the food industries
	Approach and target: Voluntary: The Community Nutrition Promotion Sector has approached six local manufacturers of fruit juices and SSBs in August 2016 with an aim to reduce the AS in their products. The producers have shown great support to gradually reduce the sugar content in the nectars and drinks considering that they are the main source of FS intake among children and adults [187]. The sugar reduction initiative included biscuits and cakes as well.	Approach: Consumer awareness and education (being finalized).	Approach: Mandatory: Nutrition labeling of all prepackaged food products except for raw products such as fresh fruits, vegetables, meat and fish (including % of daily intake) [188].	Approach: Motivate food industries, via a collaboration between the Community Nutrition Promotion Sector and the Ministry of Education, in order to improve the school canteen food options, so that only the Nectar products with reduced sugar content are allowed into the school canteens
				Setting: Schools [187].
		Information provided by the NFP		
	Name of initiative: National strategy for the prevention and response to chronic noncommunicable diseases in the State of Kuwait 2017–2025	Name of initiative: Nutrition and health education programs		Name of initiative: National Committee for Nutrition promotion of school children
	Year: 2017	Year: NA		Year: 2021
	Leadership: Led by the government	Leadership: Led by the government		Leadership: Led by the PAFN
	Approach and target: Reduction of free and AS content in foods and beverages (not adopted) [189].	Approach: Mass and social media awareness programs (campaigns, events).		Approach: Voluntary guidelines, education and procurement policy
				Setting: Schools.
		Information provided by the NFP		Information provided by the NFP
			Name of initiative: Implementing the FOPL	Name of initiative: Obesity Prevention Project
			Year: 2018	Year: 2022
			Leadership: Led by the government	Leadership: Led by the PAFN
			Approach: Voluntary: Traffic Light labeling standard (Proposal submitted and awaiting approval from related technical committee).	Approach: Voluntary guidelines, education and procurement policy
				Setting: Schools.
			Information provided by the NFP	Information provided by the NFP
				Name of initiative: NA
				Year: NA
				Leadership: NA
				Approach: Prohibited the marketing of soft drinks and sweet biscuits
				Setting: Schools [167].
Lebanon	-	Name of initiative: Guideline to limit the intake of sugar, especially AS from sweetened foods and beverages	Name of initiative: General Standard for the Labeling of Pre-packaged Foods (NL 206:2017) and Guidelines for Use of Nutrition and Health Claims (NL 661:2017)	Name of initiative: NA
		Year: 2013	Year: 2017	Year: NA
		Leadership: Led by the American University of Beirut	Leadership: Led by the government	Leadership: NA
		Approach: Inclusion in FBDG [190].	Approach: Mandatory sugar claims for all food categories, whenever applicable [191,192].	Approach: Prohibited the marketing of soft drinks and sweet biscuits
				Setting: Schools [167].
Libya	-	Name of initiative: Guideline for reducing the consumption of foods high in AS	-	-
		Year: 2020		
		Leadership: Led by the National Center for Disease Control		
		Approach: Inclusion in the Dietary Guidelines for Chronic Diseases [193].		
Morocco	Name of initiative: National Health Plan 2025	Name of initiative: National Health Plan 2025	Name of initiative: Labeling of food products (n°6684 of 21/06/2018)	Name of initiative: Dietary and health guidelines for the preparation of menus in school canteens and boarding schools
	Year: 2018	Year: 2018	Year: 2018	Year: 2013
	Leadership: Led by the government (MOH)	Leadership: Led by the government (MOH)	Leadership: Led by the government (Ministry of Agriculture and MOH)	Leadership: Led by the government (MOH)
	Approach and target: Encourage the food industry to lower the sugar levels in their products [194].	Approach and target: Encourage citizens, in particular children and poor populations, to consume less sugary products, less taxed, cheaper and healthier products [194].	Approach: Mandatory: Labeling of food products with sugar and percentage of daily intake. Voluntary: FOPL (awaiting implementation) [195].	Approach: Limiting the availability of the following foods: jams, candies, chocolates, cookies, cakes, ice cream, sweet breads, croissants, pancakes, cornflakes containing AS, flavored water, soft drinks and sodas, sweetened tea, coffee and infusions
				Setting: Schools, boarding schools and universities (not adopted) [196,197].
	A proposal was developed in 2022 to set standards for agri-food products (Biscuits, pastries, milk and dairy products, cereals, confectionery, chocolate, candy, chewing gum, prepared broths, prepared soups, syrups, sauces, fruit juices (nectar, drinks), flavored drinks) regarding their sugar concentrations. Standards are being finalized and the draft for the finance law will be executed by 2023. The reduction will be mandatory as of year 2025 for all foods and beverages, with a reduction of 25% in AS content in processed foods as compared to year 2022. The reduction in sugar content will be as follows: 50% for dairy desserts, 20% for yogurt drinks, 40% for sweetened condensed milk, 40% for cookies, 29% for confectionery, 34% for chocolates and 33% for jam [198,199].			
		Name of initiative: National Nutrition Program	Name of initiative: Nutri-score	Name of initiative: National Multisectoral Strategy for the Prevention and Control of Non-Communicable Diseases 2019–2029
		Year: 2019	Year: 2023; legislative decree being finalized	Year: 2019
		Leadership: Led by the government (MOH)	Leadership: Led by the government (MOH) and WHO	Leadership: Led by the government (MOH) and General Directorate of Customs
		Approach: Promote healthy nutrition throughout the lifecycle by reducing the consumption of sugar-rich foods and beverages [143].	Aproach: Voluntary [200].	Approach: Setting sugar standards and procurement policy, and providing education and voluntary guidelines
				Setting: Schools [140].
Oman	Name of initiative: National plan for the prevention and control of chronic non-communicable diseases 2016–2025	Name of initiative: Guideline for low consumption of sugar	Name of initiative: GSO standard for Labeling of Prepackaged Food Stuffs (GSO 9/2013 (E))	Name of initiative: National plan for the prevention and control of chronic non-communicable diseases 2016–2025; National Nutrition Strategy and Framework for Action 2020–2030
	Year: 2016	Year: 2009; being updated in 2022	Year: 2013	Year: 2016 and 2020 respectively
	Leadership: Led by the government (MOH)	Leadership: Led by the government	Leadership: Led by the GCC Standardization Organization	Leadership: Led by the government (MOH)
	Approach and target: Encourage manufacturers to produce small-sized portions of food products that are high in sugars (not adopted) [152].	Approach: Inclusion in FBDG [201].	Approach: Mandatory for all food categories: Labeling for prepacked food with detailed ingredients and nutritive value of the food, as well as percentage of daily intake (this includes TS).	Approach: Food and beverages that are high in sugar have been prohibited from being sold at schools
				Setting: Schools [151,152,167].
			Information provided by the NFP	
	Name of initiative: Policy statement for sugar reduction	Name of initiative: Promoting healthier lifestyle and wellbeing		Name of initiative: Implementing Nutrient Profile in School
	Year: 2022	Year: 2021		Year: NA (planned)
	Leadership: Led by the government (MOH)	Leadership: Led by the WHO and government (MOH)		Leadership: Led by the government
	Approach and target: Voluntary: Product reformulation (for instance, sugar in SSBs to be reduced by 20% by 2025) (planned)	Approach: awareness aimed at reducing the consumption of unhealthy food high in sugar, as well as other components (planned) [130].		Approach: Providing education and voluntary guidelines
				Setting: Schools.
	Information provided by the NFP			Information provided by the NFP
		Name of initiative: National Nutrition Campaign		
		Year: 2022–2023		
		Leadership: Led by the government, in collaboration with the WHO, FAO and Aljisr Foundation		
		Approach: An awareness campaign for the community to reduce sugar, salt and unhealthy fat; through social marketing, TV advertisements, events, lectures for community (online and in person), videos, social media posts, posters, messages in the public transport, etc.		
		Information provided by the NFP		
Pakistan	Name of initiative: National Action Plan for Reduction of Dietary Risks of NCDs for Pakistan	Name of initiative: Guideline to reduce sugar intake, and limit intake of soft drinks, confectionaries, bakery products and commercial fruit drinks	Name of initiative: National Action Plan for Reduction of Dietary Risks of NCDs for Pakistan	Name of initiative: NA
	Year: 2022 (Planned)	Year: 2019	Year: 2022 (Planned)	Year: NA
	Leadership: Led by the government (Ministry of National Health Services, Regulation and Coordination), in collaboration with the WHO	Leadership: Led by the government (Ministry of Planning, Development and Reform) and FAO UN	Leadership: Led by the government (Ministry of National Health Services, Regulation and Coordination); in collaboration with the WHO	Leadership: Led by Provincial Food Authorities of Punjab, Sindh, KP and Balochistan
	Approach and target: Mandatory for all foods and snacks (bakery products, breakfast cereals, desserts, puddings and ice cream, SSBs, biscuits and cakes, ultra-processed foods): Approach includes: (1) targets for sugar levels in foods and snacks and (2) revision of standards through Pakistan Standards and Quality Control Authority and Provincial Food Authorities.	Approach: Inclusion in Pakistan Dietary Guidelines for Better Nutrition [202].	Approach: Warning labels; Mandatory for certain food categories such as “foods high in sugar”.	Approach: Restrictions on availability of SSBs and energy drinks, and foods high in sugar/ultra-processed foods, in schools/educational institutions; traffic lights have been introduced (foods in red category are not allowed in the premises of the school and within the 100-m radius of the educational institutions, similarly the availability and consumption of food in green category is encouraged in the premises of the school)
				Setting: Schools/educational institutions [203].
	Information provided by the NFP		Information provided by the NFP	
		Name of initiative: Saving Youth from Sugar-sweetened Beverages through Education, Media, and Advocacy (SYSTEM)		
		Year: 2020		
		Leadership: Pakistan National Heart Association (NGO) working closely with several government Ministries and key institutions		
		Approach: Anti sugary drinks campaign through social marketing, events and media advocacy [154].		
		Name of initiative: National Action Plan for Reduction of Dietary Risks of NCDs for Pakistan		
		Year: 2022 (Planned)		
		Leadership: Led by the government (Ministry of National Health Services, Regulation and Coordination); in collaboration with the WHO		
		Approach: Multisectoral approach; awareness raising.		
		Information provided by the NFP		
Palestine	Name of initiative: National Health Strategy 2017–2022	Name of initiative: National Health Strategy 2017–2022	-	Name of initiative: NA
	Year: 2017	Year: 2017		Year: NA
	Leadership: Led by the government (MOH) in cooperation with all health sector stakeholders	Leadership: Led by the government (MOH) in cooperation with all health sector stakeholders		Leadership: NA
	Approach and target: Mandatory technical instructions to reduce sugar in food products (to be endorsed and applied by 90%) (planned) [155].	Approach: Raising awareness to help reduce the consumption of sugar in line with the WHO (planned) [155].		Approach: Prohibited the marketing of soft drinks and sweet biscuits in schools
				Setting: Schools [167].
		Name of initiative: Guideline for reduction of sugary foods and beverages, specifically AS		
		Year: 2021		
		Leadership: Led by the government (MOH)		
		Approach: Inclusion in FBDG [204].		
Qatar	Name of initiative: Qatar National Nutrition and Physical Activity Action Plan 2017–2022	Name of initiative: Start now campaign		Name of initiative: Food safety and health guide
	Year: 2017	Year: 2017		Year: 2016
	Leadership: Led by the government (MOPH)	Leadership: Led by the government (MOPH)		Leadership: Led by the government (Ministry of Education and Higher Education Department of Health and Safety)
	Approach and target: Progressive reformulation of sugar-rich drinks and foods (not adopted) [156].	Approach: Encourages the consumption of wholegrains, vegetables, fish and chicken, and discourages foods high in salt, sugar and fat [205].		Approach: EI from TS in school meals should be limited to 35% (or 17.5 g) per person
				Setting: Schools (not adopted) [206,207].
	Name of initiative: Initiative to reduce fat, sugar and salt consumption in Qatar	Name of Initiative: Smart Start Campaign		Name of initiative: Guidance for supervisors of school canteens for the academic year 2018–2019
	Year: 2019	Year: 2019		Year: 2018
	Leadership: Led by the government (MOPH)	Leadership: Qatar University		Leadership: Led by the government (Ministry of Education and Higher Education Department of Health and Safety)
	Approach: Voluntary: Approach done through meetings with the food industries (dairy beverages, bread, pastries, baked goods, bottled juices) to reformulate their products and reduce the amount of sugar used during manufacturing.	Approach: Promotes the importance of physical and psychological health of children aged 3–6 years; included sugar awareness on the dangers of excess sugar intake [208].		Approach: Food items prohibited from being sold at schools; they include desserts and sweets (jellies, lollipops, chewing gum), carbonated beverages, ice cream, chocolates, energy drinks, milk and yogurt that contain more than 22 g and 30 g of sugar/240 mL,
				Setting: Schools [167,206,207,209].
	Information provided by the NFP			
		Name of initiative: Initiative to reduce fat, sugar and salt consumption in Qatar		Name of initiative: Food & Beverage Guidelines; School Canteen Guidelines; Educational sessions in schools and workplaces
		Year: 2019		Year: Ongoing
		Leadership: Led by the government (MOPH)		Leadership: Led by the government (MOPH)
		Approach: Social marketing (e.g., campaigns, TV advertising (conducted TV and radio interviews), events.		Approach: Education, procurement policy, voluntary guidelines. School Canteen Guidelines are mandatory in governmental schools
				Setting: Schools, hospitals, workplace.
		Information provided by the NFP		Information provided by the NFP
	-	Name of initiative: Guideline for low consumption of sugar		-
		Year: 2015		
		Leadership: Led by the Supreme Council of Health		
		Approach: Inclusion in FBDG [210].		
Somalia		Name of Initiative: Somalia Nutrition Strategy (2020–2025)	-	
		Year: Target by 2025		
		Leadership: Ministry of Health and Human Services, Federal Republic of Somalia		
		Approach and Target: Providing comprehensive and routine nutritional assessment and counseling services to adolescents at communities, schools and health facilities with the goal of reducing the intake of FS in children and adult meals and reducing the consumption of SSBs [211].		
Sudan	-	Name of initiative: National Nutrition Strategic Plan 2014–2025	-	-
		Year: 2014		
		Leadership: Led by the government (MOH)		
		Approach: Promote nutritional knowledge and appropriate attitudes and practices of caregivers towards food, social and dietary customs, family/child care and feeding practices; promote low sugar intake among the public (not adopted) [212].		
Tunisia	Name of initiative: National Obesity Control and Prevention	-	Name of initiative: NA	-
	Year: 2017		Year: 2008	
	Leadership: Led by the government (Ministry of Commerce) with the cooperation of industries		Leadership: Led by the government (Ministry of Commerce and Crafts)	
	Approach and target: Reduction of sugar content in beverages, milk and milk products [118,120,158].		Approach: The amount of sugar should be indicated on the label. Nutrition claims indicating “sugar-free” should not contain more than 0.5 g of sugars per 100 g or per 100 mL (not adopted) [213].	
UAE	Name of initiative: National Action Plan in Nutrition; National Plan for Prevention and Response to NCDs 2017–2021	Name of initiative: National Plan to Combat Childhood Obesity United Arab Emirates	Name of initiative: Weqaya Food Program (ADS13/2018)–health logo scheme in Abu Dhabi	Name of initiative: Guideline to Healthy and Nutritious Food Practices in School Canteens–Dubai
	Year: 2017	Year: 2017	Year: 2015	Year: 2011
	Leadership: Led by the government (MOHAP)	Leadership: Led by the government (MOHAP–Department of Health Education and Promotion)	Leadership: Led by Abu Dhabi Quality and Conformity Council	Leadership: Led by Dubai Municipality–Dubai Health Authority
	Approach and targets: Regulation of the maximum limit of sugar in SSBs (8 g/100 mL i.e., 2 teaspoons) (in process of submission to Higher Authorities for endorsement) [161,162].	Approach: Limit the consumption of foods and beverages high in sugar among infants and young children by providing guidance and support to caregivers regarding foods to be avoided (such as sugar- sweetened milks, fruit juices or energy-dense and nutrient poor foods) (not adopted) [159].	Approach: Voluntary: Logo on foods/dishes or meals should meet certain AS levels in meals/takeaway food, children’s meals, or takeaway food and individual food items [173,214].	Approach: Guideline provided to the school canteens and staff regarding foods that are not allowed (soft drinks, energy drinks, all types of fruit drinks, milk and yogurt with artificial flavors, chewing gum, candies, sweets, chocolates). Moreover, AS content should not exceed 6% in food preparations and sugars should be limited to 2% of daily recommended allowance in packaged products
				Setting: Schools (not adopted) [215].
		Name of initiative: Guideline for low consumption of sugar	Name of initiative: National Program for Happiness and Wellbeing	Name of initiative: School Canteen Guidelines of the Emirate of Abu Dhabi
		Year: 2019	Year: 2020	Year: 2011
		Leadership: Led by the government (MOHAP), in collaboration with other health authorities, government-related sectors, academia and municipalities	Leadership: Led by the government (MOH and the Standards and Metrology Organization)	Leadership: Abu Dhabi Education Council, Abu Dhabi Food Control Authority and Health Authority
		Approach: Inclusion in FBDG [216].	Approach: Voluntary FOP traffic light labeling for sugar levels in prepackaged foods; to become mandatory in 2022 [173,217].	Approach: TS and AS of products offered in schools should not exceed 35% of the product’s weight
				Setting: Schools (not adopted) [218].
				Name of initiative: National Plan to Combat Childhood Obesity United Arab Emirates
				Year: 2017
				Leadership: Led by the government (MOHAP–Department of Health Education and Promotion)
				Approach: Eliminate the provision and sale of unhealthy foods (SSBs and energy-dense, nutrient-poor foods)
				Setting: Schools (not adopted) [159].
				Name of initiative: National Action Plan in Nutrition
				Year: 2017
				Leadership: Led by the government (MOHAP)
				Approach: Reduce sugar consumption
				Setting: Prisons, juvenile centers, police and military schools, army forces, geriatric home cares and their canteen [161].
				Name of initiative: Department of Health Guideline for Vending Machines and Retail Items in Health Care Facilities–Abu Dhabi
				Year: 2021
				Leadership: Led by the government (Department of Health)
				Approach: A healthy vending machine should not contain foods high in AS (soft drinks, sports drinks, energy drinks, chocolates except for dark, sugar candies); packed snacks should not exceed 35% of total weight from sugar (i.e., a maximum of 20 g of TS)
				Setting: Health care facilities [219].

Abbreviations: AS, added sugars; CNPS, Community Nutrition Promotion Sector; EI, energy intake; EMR, Eastern Mediterranean Region; FAO, Food and Agriculture Organization; FBDG, food-based dietary guidelines; FNA, Food and Nutrition Authority; FOP, front-of-pack; FOPL, front-of-pack labeling; FS, free sugars; GCC, Gulf Cooperation Council; GSO, GCC Standardization Organization; KP, Khyber Pakhtunkhwa; KSA, Kingdom of Saudi Arabia; MOH, Ministry of Health; MOHAP, Ministry of Health and Prevention; MOPH, Ministry of Public Health; NA, not available; NCD, non-communicable disease; NFP, nutrition focal point; NGO, non-governmental organization; PAFN, Public Authority for Food and Nutrition; SFDA, Saudi Food and Drug Authority; SSB, sugar-sweetened beverages; TFA, trans fatty acids; TOT, training of trainers, TS, total sugars; TV, television; UAE, United Arab Emirates; UN, United Nations; WHO, World Health Organization; WHO EMRO: World Health Organization Eastern Mediterranean Regional Office.

**Table 3:** National sugar reduction strategies or action plans identified in the EMR countries.

Country	National Strategy and/or Action Plan
Jordan	Reduction of FS intake in the population, specifically among children and adolescents, to <10% of EI–2015 (MOH) (National Strategy And Plan Of Action Against Diabetes, Hypertension, Dyslipidemia And Obesity in Jordan) (not adopted) [220]
KSA	Reduction of monosaccharides consumption by 10% in the coming 10 years (an average of 1% per year)–2017 (MOH) (National Obesity Control Program) (not adopted) [221] and 2014 (Government of KSA) (National Executive Plan for NCDs 2014–2025) (not adopted) [222]
	Reduction in consumption of products rich in sugars to less than 10% of total EI and to less than 5% for more health benefits–2015 (MOH) (National Strategy for Healthy Food and Physical Activity 2015–2025) (not adopted) [223] and 2014 (Government of KSA) (KSA National Strategy for Diet and Physical Activity for the Years 2014–2025) (not adopted) [224]
Morocco	Reduction in population sugar intake–2015 (MOH) (Prevention of NCDs: Multisectoral Action Plan for a Healthy Lifestyle 2015–2020) [225]
Oman	Reduction in sugar intake by 20% by 2025 (MOH) (National Nutrition Strategy) (Information provided by the NFP)
	Reduction in population sugar intake, gradually to reach 100% by 2025–2016 (MOCI) (National plan for the prevention and control of chronic non-communicable diseases 2016–2025) [152]

Abbreviations: EI: energy intake; EMR: Eastern Mediterranean Region; FS: free sugars; KSA: Kingdom of Saudi Arabia; MOCI: Ministry of Commerce and Industry; MOH: Ministry of Health; NCDs: noncommunicable diseases; NFP: nutrition focal point.

## Data Availability

Not applicable.

## Supplementary Materials

The following are available online at https://www.mdpi.com/article/10.3390/nu15010055/s1, Table S1: PRISMA Checklist, Table S2: Example of a Database Search, Table S3: Estimates of TS Intakes in Countries of the EMR; Table S4: Estimates of AS Intakes in Countries of the EMR, Table S5: Estimates of FS Intakes in Countries of the EMR, Table S6: Sugar Levels in Food and Meals, Table S7: Knowledge, Attitudes and Behavior (KAB) towards Sugar in countries of the EMR. Reference [252] is cited in the supplementary materials

## Abbreviations

AS: added sugars; AWRS: Arab World Research Source; CNPS: Community Nutrition Promotion Sector; CVD: cardiovascular diseases; EFSA: European food safety agency; EI: energy intake; EMR: Eastern Mediterranean Region; EMRO: Regional Office for the Eastern Mediterranean; FAO: Food and Agriculture Organization; FBDG: food-based dietary guidelines; FNA: Food and Nutrition Authority; FDA: Food and Drug Administration; FOP: front of pack; FOPL: front of pack labelling; FS: free sugars; GCC: Gulf Cooperation Council; GINA: Global Database on the Implementation of Nutrition Action; GSO: GCC Standardization Organization; GZAT: General Authority of Zakat, Tax and Customs; HIES: Households Income and Expenditure Survey; IASJ: Iraqi Academic Scientific Journals; IOM: Institute of Medicine; JFDA: Jordanian Food and Drug Administration; KAB: knowledge, attitudes and behavior; KP: Khyber Pakhtunkhwa; KSA: Kingdom of Saudi Arabia; MOCI: Ministry of Commerce and Industry; MOF: Ministry of Finance; MOH: Ministry of Health; MOHAP: Ministry of Health and Prevention; MOPH: Ministry of Public Health; NA: not available; NCD: non-communicable disease; NFP: nutrition focal point; NGO: non-governmental organization; PAFN: Public Authority for Food and Nutrition; SFDA: Saudi Food and Drug Authority; SSBs: sugar-sweetened beverages; TOT: training of trainers; TS: total sugars; TV: television; UAE: United Arab Emirates; UK: United Kingdom; UN: United Nations; US: United States; WHO: World Health Organization.

## Appendix A

**Table A1:** List of search terms.

Sugar	sugar* OR sucrose OR syrup* OR beverage* OR cola OR drink* OR soda OR sweet* OR chocolate* OR “ice cream*” OR candy OR candies OR cookie* OR jam OR confectioner* OR confectionar* OR cake* OR jelly OR jellies OR pastries OR pastry OR biscuit* OR bakery OR bakeries OR juice* OR honey OR pie OR pies OR dessert* OR cacao OR jam OR “chewing gum*” OR liquorice* OR marmalade* OR SSB OR cereal* OR smoothies OR milkshake* OR molasses OR fructose OR glucose
AND	
Reduction OR Intake	(reduce* OR reduction* OR reducing OR decreas* OR limit OR limits OR limitation* OR limiting OR restrict* OR reformulat* OR low*) OR (consumption OR consuming OR consume OR consumes OR intake* OR food* OR nutrition OR diet* OR source*)
AND	
Strategy/policy	standard* OR polic* OR initiative* OR tax* OR program* OR regulation* OR strateg* OR guideline* OR practice* OR legislat* OR action* OR plan OR plans OR intervention* OR law* OR campaign* OR marketing OR advertise* OR label* OR incentive* OR ban* OR recommendation* OR subsidy OR subsidies OR fiscal OR levy OR levies OR levied OR price* OR pricing OR excise* OR fee OR fees OR fine OR fines OR cost*
AND	
EMR	Afghan* OR Bahrain* OR Iran* OR Persia* OR Iraq* OR Jordan* OR Kuwait* OR Lebanon* OR Lebanese OR Libya* OR Oman* OR Palestin* OR Gaza* OR “West Bank” OR Qatar* OR Saud* OR KSA OR Syria* OR Tunis* OR “United Arab Emirate*” OR UAE OR Djibouti* OR Egypt* OR Morocc* OR Pakistan* OR Somal* OR Sudan* OR Yemen* OR Levant* OR “East* Mediterranean” OR Gulf OR GCC OR Arab OR Arabia OR Arabs OR EMR OR “Middle East*” OR MENA OR “North* Africa*” OR “East* Africa*” OR “Near East*” OR “Abu Dhabi” OR Dubai OR Ajman OR Fujaira* OR Sharja* OR *Khaima* OR *Qaiwain OR *Quwain

Abbreviations: EMR: Eastern Mediterranean Region; KSA: Kingdome of Saudi Arabia; UAE: United Arab Emirates. *: Refers to a technique (known as truncation) used in research when searching databases, in order to broaden the search and ensure the inclusion of the various word endings and spelling.
