# Supplementary material for: Sugar Reduction Initiatives in the Eastern Mediterranean Region: A Systematic Review
Source: Nutrients. 2022 Dec 22;15(1):55. doi: 10.3390/nu15010055 (PMC9823488; doi:10.3390/nu15010055)
Supplement: Supplementary file 1 [file nutrients-15-00055-s001.zip › nutrients-2087094-supplementary/Table S2.pdf]

Table S2. MEDLINE (ovid) search.

Search Number	Query	Results
1	exp Dietary Sugars/	5233
2	exp Sucrose/	31691
3	exp Beverages/	143172
4	Sugars/	1994
5	fructose/ or glucose/	168993
6	Molasses/	1097
7	exp candy/ or chocolate/ or honey/	8504
8	Cacao/	3311
9	Ice Cream/	668
10	(sugar* or sucrose or fructose or glucose or molasses).ti,ab.	653858
11	(syrup* or beverage* or cola or drink* or soda or juice* or smoothie* or milkshake*).ti,ab.	203363
12	ice cream.ti,ab.	1384
13	chewing gum.ti,ab.	1866
14	(sweet* or chocolate* or candy or candies or cookie* or jam or confectioner* or confectionar* or cake* or jelly or jellies or pastries or pastry or biscuit* or bakery or bakeries or honey or pie or pies or dessert* or cacao or cocoa or liquorice* or marmalade* or SSB or cereal*).ti,ab.	99293
15	1 or 2 or 3 or 4 or 5 or 6 or 7 or 8 or 9 or 10 or 11 or 12 or 13 or 14	1071946
16	(reduce* or reduction* or reducing or decreas* or limit or limits or limitation* or limiting or restrict* or reformulat* or low or lower* or consumption or consuming or consume or consumes or intake* or food* or nutrition or diet* or source*).ti,ab.	10066913
17	exp Taxes/	10735
18	Government Programs/	5564
19	exp Nutrition Policy/	11496
20	Legislation, Food/	2451
21	(standard* or polic* or initiative* or tax* or program* or regulation* or strateg* or guideline* or practice* or legislat* or action* or plan or plans or intervention* or law or laws or campaign* or marketing or advertis* or label* or incentive* or ban or bans or banning or recommendation* or subsidy or subsidies or fiscal or levy or	7666358

	levies or levied or price* or pricing or excise* or fee or fees or fine or fines or cost*).ti,ab.	
22	17 or 18 or 19 or 20 or 21	7675408
23	africa, northern/ or egypt/ or libya/ or morocco/ or tunisia/ or djibouti/ or somalia/ or south sudan/ or sudan/ or middle east/ or afghanistan/ or bahrain/ or iran/ or iraq/ or jordan/ or kuwait/ or lebanon/ or oman/ or qatar/ or saudi arabia/ or syria/ or united arab emirates/ or yemen/ or pakistan/ or africa, eastern/	136301
24	(Afghan* or Bahrain* or Iran* or Persia* or Iraq* or Jordan* or Kuwait* or Lebanon* or Lebanese or Libya* or Oman* or Palestin* or Gaza* or "West Bank" or Qatar* or Saud* or KSA or Syria* or Tunis* or "United Arab Emirate*" or UAE or Djibouti* or Egypt* or Morocc* or Pakistan* or Somal* or Sudan* or Yemen* or levant* or "East* Mediterranean" or Gulf or GCC or Arab or Arabia or Arabs or EMR or "Middle East*" or MENA or "North* Africa*" or "East* Africa*" or "Near East*" or "Abu Dhabi" or Dubai or Ajman or Fujaira* or Sharja* or Khaima* or Qaiwain or Quwain).ti,ab.	261721
25	23 or 24	290835
26	15 and 16 and 22 and 25	3838
27	limit 26 to yr="1995 -Current"	3642
