# Supplementary material for: Sugar Reduction Initiatives in the Eastern Mediterranean Region: A Systematic Review
Source: Nutrients. 2022 Dec 22;15(1):55. doi: 10.3390/nu15010055 (PMC9823488; doi:10.3390/nu15010055)
Supplement: Supplementary file 1 [file nutrients-15-00055-s001.zip › nutrients-2087094-supplementary/Table S3.pdf]

Table S3. Estimates of TS Intakes in Countries of the EMR.

Country	Reference	Year	National or Regional	Method used	Study Population	Estimated TS intake
**Afghanistan**	Central Statistics Organization 2014 [1]	2011-2012	National	7-day food consumption recall	0-85+ year olds; n=21000	% contribution to EI: 5%
**Bahrain**	Gharib and Rasheed 2011 [2]; Cross-sectional	1999-2001	National	24-hr recall	Children and adolescents aged 6-18 years; n=2562	Mean daily intake: 101.3 ± 37.3 g among males and 89.1 ± 36.1 g among females
**Egypt**	Brouzes et al 2020 [3]; Descriptive, cross sectional	2016/2017	Regional; 4 urban areas (Greater Cairo, Alexandria, Delta and Upper Egypt)	4-day food diary	Adults women aged 19-30 years; N=130	Mean daily intake: 98 ± 54 g<br><br>% contribution to EI: 16%
**Iran**	Eini-Zinab et al 2018 [4]	2016-2018	National	Households Income and Expenditure Survey	Households; n= 100500	Daily usual consumption: 81.6 g
	Naeeni et al 2014 [5]; Cross-sectional	2009-2010	Regional; Isfahan	24-hr recall food frequency	School and junior high school pupils; n=4700	Mean daily intake: 1.3 ± 1.1 g
	Montazerifar et al 2012 [6]; Cross-sectional	-	Regional; 8 cities in Sistan	2-day 24-hr recalls and FFQ	High school girls aged 14-18 years; n=753	Mean daily intake: 95.6 ± 23.7 g
	Fami et al 2002 [7]	-	Regional; 40 villages in Tafresh area	24-hr recalls	Rural women; n=75	% contribution to EI: 7.6%
**Jordan**	Alkurdi et al 2011 [8]	2006-2007	National	Households Income and Expenditure Survey	Households; n=12768	Mean yearly consumption: 33.5 kg/person; 358 kcal/person/day (11.8% EI)
	Takruri et al 2021 [9]; Cross-sectional	2018	Regional; Amman	3-days food record	Adolescents aged 14-18 years from private and public schools; n=398	School canteens:<br>- Sugar intake: 43 ± 3 g/day (48% EI) among males vs. 35 ± 2 g/day (45% EI) among females<br><br>Markets nearby schools:

						- Sugar intake: 37 ± 3 g/day (42% EI) among males vs. 34 ± 3 g/day (44% EI) among females
	Thana'Y et al 2020 [10]; Cross-sectional	2018	Regional; Amman	3-days food record	Adolescents aged 14-18 years from private and public schools; n=398	Mean daily intake: 88.8 ± 2.9 g among males and 77.9 ± 3.1 g among females
**KSA**	Collison et al 2010 [11]; Cross-sectional	2007	Regional; Riyadh, different regions	FFQ	10-19 year old school children from 450 schools; n=9433	Weekly mean intakes: 172.47 ± 84.93 g<br><br>Weekly % contribution to EI: 26.3% of total energy
**Lebanon**	Jomaa et al 2022 [12]; Cross-sectional	2012	National	24-hr recall	Underfive children; n=866	Mean daily intake:<br>- 0-5.9 months: 60.7 ± 1.5 g<br>- 6-11.9 months: 69.7 ± 2.4 g<br>-12-23.9 months: 74.9 ± 2.2 g<br>- 24-47.9 months: 76.8 ± 1.8 g<br><br>% contribution to EI:<br>- 0-5.9 months: 40 ± 0.3%<br>- 6-11.9 months: 31.8 ± 0.6%<br>-12-23.9 months: 25.6 ± 0.5%<br>- 24-47.9 months: 21.1 ± 0.4%
	Nasreddine et al 2022 [13]; Cross-sectional	2014-2015	National	Based on the Lebanese Food and Nutrition Security Survey (L-FANUS); 24-hr recalls	Children and adolescents aged 4-13 years; n=771	Mean daily intake: 77.6 ± 1.8 g<br><br>% contribution to EI: 17.4 ± 0.3%
	Jomaa et a 2021 [14]; Cross-sectional	2012 for underfive children and 2014 for those aged 6 years and above	National	24-hr recall	Underfive children, children and adolescents; n=899 underfive children and 1133 children and adolescents aged 5 years and above	Median % contribution to EI:<br>-Underfive children: 23.5%<br>- 5-18 year olds: 17.2%

	Nasreddine et al 2020 [15]; Cross-sectional	2008/2009	National	24-hr recall	Children and adolescents aged 6-19.9 years; n=956	<u>% contribution to EI:</u> 12.01 ± 0.27% (11.63 ± 0.38% among males and 12.38 ± 0.38% among females)
	Nasreddine et al 2020 [15]; Cross-sectional	2008/2009	National	24-hr recall	Adults aged 20 years and above; n=2680	<u>% contribution to EI:</u> - Adults 20-59.9 years: 10.85 ± 0.18% (10.69 ± 0.3% among males and 11.01 ± 0.26% among females) - <u>Adults 60 years and above:</u> 10.72 ± 0.4% (11 ± 0.73% among males and 10.45 ± 0.75% among females)
	Nasreddine et al 2019 [16]; Cross-sectional	1997 vs. 2008/2009	National	24-hr recall	Adolescents aged 20 years and above; n=1063 in 1997 and 2518 in 2008/2009	<u>% contribution to EI:</u> - In 1997: 11.48 ± 0.26% (10.88 ± 0.42% among males and 11.97 ± 0.34% among females) - In 2008/2009: 10.83 ± 0.17% (10.5 ± 0.26% among males and 11.07 ± 0.23% among females)
	Mansour et al 2019 [17]; Cross-sectional	2009	National	Based on the WHO Nutrition and Non-Communicable Diseases Risk Factor cross-sectional household survey conducted in Lebanon; FFQ	Adults aged 18 years and above; n=363	<u>% contribution to EI:</u> 5.7%
	Hamamji 2018 [18]; Cross-sectional	2012 for underfive children and 2014 for those aged 6 years and above	National	24-hr recall	Underfive children, children and adolescents; n=888 underfive children and 1106 children and adolescents aged 6 years and above	<u>% contribution to EI:</u> - Underfive: 20.6-27.9% - 6-18 year olds: 16.4-17.4%

	Aoun et al 2022 [19]; Cross-sectional	2014	Regional; Beirut	FFQ	Adults; n=283	Mean daily intake:<br>- TS: 104.99 ± 58.45 g<br>- Total fructose: 51.42 ± 35.54 g (6.58 ± 3.71% EI)<br>- Natural and added fructose: 12.29 ± 8.57 g and 39.12 ± 34.1 g (1.78 ± 1.41% EI and 4.80 ± 3.56% EI)
	Shatila et al 2021 [20]	2017	Regional; American University of Beirut	24-hr recall	Adults aged 18-65 years; n=62	% contribution to EI:<br>14.7 ± 4.7%
**Libya**	Huew et al 2014 [21]	-	Regional; Benghazi	3 days food diary	Adolescents aged 12 years; n=180	% contribution to EI:<br>20.4%
**Morocco**	Benjelloun 2002 [22]	1984-1985	National	Survey	Adults aged 20 years and above; n=41526	Mean yearly intake:<br>27 kg/person
**Pakistan**	Government of Pakistan 2020 [23]	2015-2016 and 2018-2019	National	Households Income and Expenditure Survey	Households in urban and rural areas in Pakistan; n=24238 in 2015-2016 and n=27062 in 2018-2019	Mean yearly intake:<br>1.42 kg/person in 2015-2016 and 1.35 kg/person in 2018-2019
**Palestine**	Stene et al 1999 [24]; Cross-sectional	1996-1997	Regional; Palestinian West Bank village	11-item qualitative FFQ and 24-hr recall	Adults aged 30-65 years; n=500	% contribution to EI:<br>13-14%<br><br>Mean household sugar consumption:<br>37.8 kg/consumption unit/year
**Sudan**	MOH and WHO [25]	2016	National	STEPS questionnaire	Adults aged 18 to 69 years	- 6.3 teaspoons were consumed weekly, on average (6.5 teaspoons among males vs. 6.2 teaspoons among females)<br><br>Rural vs. urban:<br>- 6.5 teaspoons were consumed weekly, on average, in rural

						areas (6.7 teaspoons among males vs. 6.4 teaspoons among females) vs. 6 teaspoons in urban areas (6.1 teaspoons among males vs. 5.9 teaspoons among females)
**UAE**	Nasreddine et al 2022 [26]; Cross-sectional	2019-2020	Regional; 3 major emirates: Abu Dhabi, Dubai and Sharjah	24-hr recall	Children under 4 years from hospitals' outpatient clinics and primary healthcare centers; n=525	<u>Mean daily intake:</u><br>- 0-5.9 months: 54.5 ± 1.8 g<br>- 6-11.9 months: 49.9 ± 3.4 g<br>- 12-23.9 months: 54.5 ± 3.0 g<br>- 24-35.9 months: 56.4 ± 1.7 g<br>- 36-47.9 months: 60.1 ± 1.7 g<br><br><u>% contribution to EI:</u><br>- 0-5.9 months: 33.5 ± 0.9%<br>- 6-11.9 months: 24.1 ± 0.4%<br>- 12-23.9 months: 22.3 ± 1.0%<br>- 24-35.9 months: 20.1 ± 0.6%<br>- 36-47.9 months: 20.0 ± 0.5%

Abbreviations: EI: energy intake; EMR: Eastern Mediterranean Region; FFQ: food frequency questionnaire; KSA: Kingdom of Saudi Arabia; TS: total sugars; UAE: United Arab Emirates; WHO: World Health Organization.

References

1. Central Statistics Organization, *The national risk and vulnerability assessment 2011-2012 (Afghanistan living conditions survey)*. 2014: Afghanistan.
2. Gharib, N. and P. Rasheed, *Energy and macronutrient intake and dietary pattern among school children in Bahrain: a cross-sectional study.* Nutrition Journal, 2011. **10**: p. 12.
3. Brouzes, C.M.C., et al., *Urban Egyptian Women Aged 19-30 Years Display Nutrition Transition-Like Dietary Patterns, with High Energy and Sodium Intakes, and Insufficient Iron, Vitamin D, and Folate Intakes.* Current Developments in Nutrition, 2020. **4**(2): p. 10.
4. Eini-Zinab, H., S.R. Sobhani, and A. Rezazadeh, *Designing a healthy, low-cost and environmentally sustainable food basket: An optimisation study.* Public Health Nutrition, 2020.
5. Naeeni, M.M., et al., *Nutritional knowledge, practice, and dietary habits among school children and adolescents.* International Journal of Preventive Medicine, 2014. **5**: p. S171-S178.
6. Montazerifar, F., M. Karajibani, and A.R. Dashipour, *Evaluation of dietary intake and food patterns of adolescent girls in sistan and baluchistan province, Iran.* Functional Foods in Health and Disease, 2012. **2**(3): p. 62-71.
7. Fami, H.S., V. Veerabhadraiah, and K.G. Nath, *Nutritional status of rural women in relation to their participation in mixed farming in the Tafresh area of Iran.* Food and Nutrition Bulletin, 2002. **23**(3): p. 321-329.
8. ا.ح. رباح, اتجاهات استهلاك الطاقة و العناصر الغذائية الكبرى و مصادرها الغذائية في الأردن. مجلة الجمعية السعودية للغذاء و التغذية, and أحمد, ا.ر., ف.م.ا. عزت, 6 ،2011(2): p. 40-54.
9. Takruri, H., T. ALjaraedah, and R. Tayyem, *Food and nutrient intakes from school canteens and markets nearby schools among students aged 14-18 in Jordan.* Nutrition & Food Science, 2021.
10. Thana'Y, A., R.F. Tayyem, and H.R. Takruri, *Nutrient Intakes among Jordanian Adolescents Based on Gender and Body Mass Index.* International Journal of Child Health and Nutrition, 2020. **9**(1): p. 9-16.
11. Collison, K.S., et al., *Sugar-sweetened carbonated beverage consumption correlates with BMI, waist circumference, and poor dietary choices in school children.* BMC public health, 2010. **10**(1): p. 1-13.
12. Jomaa, L., et al., *Food consumption patterns and nutrient intakes of infants and young children amidst the nutrition transition: the case of Lebanon.* Nutrition Journal, 2022. **21**(1): p. 1-15.
13. Nasreddine, L., et al., *Food and nutrient intake of school-aged children in Lebanon and their adherence to dietary guidelines and recommendations.* BMC Public Health, 2022. **22**(1): p. 1-18.
14. Jomaa, L., et al., *Dietary intakes, sources, and determinants of free sugars amongst Lebanese children and adolescents: findings from two national surveys.* European Journal of Nutrition, 2021: p. 15.
15. Nasreddine, L., et al., *Sex disparities in dietary intake across the lifespan: the case of Lebanon.* Nutrition Journal, 2020. **19**(1): p. 18.
16. Nasreddine, L., et al., *Differences in dietary intakes among Lebanese adults over a decade: Results from two national surveys 1997–2008/2009.* Nutrients, 2019. **11**(8).
17. Mansour, M., et al., *Prevalence and associations of behavioural risk factors with blood lipids profile in Lebanese adults: findings from WHO STEPwise NCD cross-sectional survey.* Bmj Open, 2019. **9**(8): p. 9.

18. Hamamji, S.E., *Intakes and sources of fat, free sugars and salt among Lebanese children and adolescents*, in *Nutrition and Food Sciences*. 2018, American University of Beirut.
19. Aoun, R., et al., *Dietary fructose and its association with the metabolic syndrome in Lebanese healthy adults: a cross-sectional study.* Diabetology & Metabolic Syndrome, 2022. **14**(1): p. 1-14.
20. Shatila, H., et al., *Impact of Ramadan fasting on dietary intakes among healthy adults: a year-round comparative study.* Frontiers in nutrition, 2021. **8**: p. 689788.
21. Huew, R., et al., *Nutrient intake and dietary patterns of relevance to dental health of 12-year-old Libyan children.* Public Health Nutrition, 2014. **17**(5): p. 1107-1113.
22. Benjelloun, S., *Nutrition transition in Morocco.* Public health nutrition, 2002. **5**(1a): p. 135-140.
23. Government of Pakistan, *Household integrated economic survey (HIES) 2018-19*. 2020, Pakistan Bureau of Statistics: Pakistan.
24. Stene, L.C.M., et al., *Food consumption patterns in a Palestinian West Bank population.* European Journal of Clinical Nutrition, 1999. **53**(12): p. 953-958.
25. Ministry of Health-Sudan; World Health Organization, *Sudan STEPwise Survey for Non-Communicable Disease Risk Factors 2016 Report*. 2016.
26. Nasreddine, L.M., et al., *Total Usual Nutrient Intakes and Nutritional Status of United Arab Emirates Children (< 4 Years): Findings from the Feeding Infants and Toddlers Study (FITS) 2021.* Current Developments in Nutrition, 2022. **6**(5): p. nzac080.
