# Supplementary material for: Sugar Reduction Initiatives in the Eastern Mediterranean Region: A Systematic Review
Source: Nutrients. 2022 Dec 22;15(1):55. doi: 10.3390/nu15010055 (PMC9823488; doi:10.3390/nu15010055)
Supplement: Supplementary file 1 [file nutrients-15-00055-s001.zip › nutrients-2087094-supplementary/Table S4.pdf]

Table S4. Estimates of AS Intakes in Countries of the EMR.

Country	Reference	Year	National or Regional	Method used	Study Population	Estimated AS intake
**Egypt**	Brouzes et al 2020 [1]; Descriptive, cross sectional	2016/2017	Regional; 4 urban areas in Egypt (Greater Cairo, Alexandria, Delta and Upper Egypt)	4-day food diary	Adults women aged 19-30 years; n=130	Mean daily intake: 25.8 ± 19.2 g of TS
**Iran**	Shahinfar et al 2020 [2]; Cross-sectional	2015-2016	Regional; Tehran	Semi-quantitative FFQ	Older adults; n=226	Mean daily intake: 151.4 ± 62.4 g
	Hashemi et al 2018 [3]; Cross-sectional	2014	Regional; Hamadan city	Semi-quantitative FFQ	Women aged 23-49 years; n=823	Mean daily intake: 140.76 ± 153.02 g<br><br>Mean contribution to energy: 255.01 ± 28.01 calories<br><br>% contribution to EI: 11.67%
**Lebanon**	Jomaa et al 2022 [4]; Cross-sectional	2012	National	24-hr recall	Underfive children; n=866	Mean daily intake:<br>- 0-5.9 months: 0.9 ± 0.3<br>- 6-11.9 months: 10.6 ± 1.4 g<br>- 12-23.9 months: 19.5 ± 1.5 g<br>- 24-47.9 months: 34.1 ± 1.6 g
	Nasreddine et al 2022 [5]; Cross-sectional	2014-2015	National	Based on the Lebanese Food and Nutrition Security Survey (L-FANUS); 24-hr recalls	Children and adolescents aged 4-13 years; n=771	Mean daily intake: 51.2 ± 1.6 g<br><br>% contribution to EI: 11.2 ± 0.3 %

**UAE**	Nasreddine et al 2022 [6]; Cross-sectional	2019-2020	Regional; 3 major emirates: Abu Dhabi, Dubai and Sharjah	24-hr recall	Children under 4 years from hospitals' outpatient clinics and primary healthcare centers; n=525	Mean daily intake:<br>- 0-5.9 months: 0.6 ± 0.2 g<br>- 6-11.9 months: 5.1 ± 0.8 g<br>- 12-23.9 months: 13.6 ± 1.4 g<br>- 24-35.9 months: 22.8 ± 0.9 g<br>- 36-47.9 months: 25.4 ± 0.9 g<br><br>% contribution to EI:<br>- 0-5.9 months: 0.3 ± 0.1%<br>- 6-11.9 months: 2.6 ± 0.4%<br>- 12-23.9 months: 5.5 ± 0.7%<br>- 24-35.9 months: 8.2 ± 0.3%<br>- 36-47.9 months: 8.5 ± 0.3%

Abbreviations: AS: added sugars; EI: energy intake; EMR: Eastern Mediterranean Region; FFQ: food frequency questionnaire; TS: total sugars; UAE: United Arab Emirates.

References

1. Brouzes, C.M.C., et al., *Urban Egyptian Women Aged 19-30 Years Display Nutrition Transition-Like Dietary Patterns, with High Energy and Sodium Intakes, and Insufficient Iron, Vitamin D, and Folate Intakes.* Current Developments in Nutrition, 2020. **4**(2): p. 10.
2. Shahinfar, H., et al., *Association of dietary energy density with cardiometabolic risk factors and metabolic syndrome in Tehranian older adults.* Journal of Cardiovascular and Thoracic Research, 2020. **12**(2): p. 97-105.
3. Hashemi, S.Z., et al., *Nutrient intake and unhealthy dietary pattern of Iranian women: a cross sectional study.* Progress in Nutrition, 2018. **20**: p. 106-118.
4. Jomaa, L., et al., *Food consumption patterns and nutrient intakes of infants and young children amidst the nutrition transition: the case of Lebanon.* Nutrition Journal, 2022. **21**(1): p. 1-15.
5. Nasreddine, L., et al., *Food and nutrient intake of school-aged children in Lebanon and their adherence to dietary guidelines and recommendations.* BMC Public Health, 2022. **22**(1): p. 1-18.
6. Nasreddine, L.M., et al., *Total Usual Nutrient Intakes and Nutritional Status of United Arab Emirates Children (< 4 Years): Findings from the Feeding Infants and Toddlers Study (FITS) 2021.* Current Developments in Nutrition, 2022. **6**(5): p. nzac080.
