# Supplementary material for: Sugar Reduction Initiatives in the Eastern Mediterranean Region: A Systematic Review
Source: Nutrients. 2022 Dec 22;15(1):55. doi: 10.3390/nu15010055 (PMC9823488; doi:10.3390/nu15010055)
Supplement: Supplementary file 1 [file nutrients-15-00055-s001.zip › nutrients-2087094-supplementary/Table S5.pdf]

Table S5. Estimates of FS Intakes in Countries of the EMR.

Country	Reference	Year	National or Regional	Method used	Study Population	Estimated FS intake
**Lebanon**	Jomaa et a 2021 [1]; Cross-sectional	2012 for underfive children and 2014 for those aged 6 years and above	National	24-hr recall	Underfive children, children and adolescents; n=899 underfive children and 1133 children and adolescents aged 5 years and above	Median % contribution to EI: - Underfive children: 8.5% - 5-18 year olds: 11.9%
	Hamamji 2018 [2]; Cross-sectional	2012 for underfive children and 2014 for those aged 6 years and above	National	24-hr recall	Underfive children, children and adolescents; n=888 underfive children and 1106 children and adolescents aged 6 years and above	% contribution to EI: - Underfive children: 6.3-11.9% - 6-18 year olds: 12.6-12.9%
	Nasreddine et al 2014 and Nasreddine et al 2006 [3, 4]; Cross-sectional	2001	Regional; Beirut and its suburbs	Food consumption survey; quantitative FFQ	Adults aged 25-54 years; n=444	% contribution to EI: 11.4%
**Libya**	Huew et al 2014 [5]	-	Regional; Benghazi	3 days food diary	Adolescents aged 12 years; n=180	% contribution to EI: 12.6%
**Tunisia**	Abassi et al 2019 [6]; Cross-sectional	2009-2010	Regional; Greater Tunis	3 day food records<br><br>Tunisian food composition databases were used to derive nutritional content	Adults aged 20-49 years from households; 1651 females and 894 males	Mean daily intake: - Total: 31.3 ± 0.4 g/1000 kcal - Males: 30.8 ± 0.7 g/1000 kcal - Females: 31.9 ± 0.5 g/1000 kcal

	Aounallah-Skhiri et al 2011 [7]; Cross-sectional	2005	Regional; 3 regions of Tunisia	Semi-quantitative FFQ	Adolescents aged 15-19 years; n=1019	Mean daily intake:<br>26.8 g/1000 kcal
**UAE**	Nasreddine et al 2022 [8]; Cross-sectional	2019-2020	Regional; 3 major emirates: Abu Dhabi, Dubai and Sharjah	24-hr recall	Children under 4 years from hospitals' outpatient clinics and primary healthcare centers; n=525	Mean daily intake:<br>- 0-5.9 months: 4.7 ± 1.3 g<br>- 6-11.9 months: 9.8 ± 2.1 g<br>- 12-23.9 months: 18.1 ± 1.5 g<br>- 24-35.9 months: 29.5 ± 1.0 g<br>- 36-47.9 months: 31.2 ± 0.9 g<br><br>% contribution to EI:<br>- 0-5.9 months: 2.3 ± 0.5%<br>- 6-11.9 months: 4.4 ± 0.7%<br>- 12-23.9 months: 7.4 ± 0.5%<br>- 24-35.9 months: 10.6 ± 0.3%<br>- 36-47.9 months: 10.5 ± 0.3%

Abbreviations: EI: energy intake; EMR: Eastern Mediterranean Region; FFQ: food frequency questionnaire; FS: free sugars; UAE: United Arab Emirates.

References

1. Jomaa, L., et al., *Dietary intakes, sources, and determinants of free sugars amongst Lebanese children and adolescents: findings from two national surveys.* European Journal of Nutrition, 2021: p. 15.
2. Hamamji, S.E., *Intakes and sources of fat, free sugars and salt among Lebanese children and adolescents*, in *Nutrition and Food Sciences*. 2018, American University of Beirut.
3. Nasreddine, L., et al., *Food consumption patterns in an adult urban population in Beirut, Lebanon.* Public Health Nutr, 2006. **9**(2): p. 194-203.
4. Nasreddine, L., et al., *Trends in nutritional intakes and nutrition-related cardiovascular disease risk factors in Lebanon: The need for immediate action.* Journal Medical Libanais, 2014. **62**(2): p. 83-91.
5. Huew, R., et al., *Nutrient intake and dietary patterns of relevance to dental health of 12-year-old Libyan children.* Public Health Nutrition, 2014. **17**(5): p. 1107-1113.
6. Abassi, M.M., et al., *Gender inequalities in diet quality and their socioeconomic patterning in a nutrition transition context in the Middle East and North Africa: a cross-sectional study in Tunisia.* Nutr J, 2019. **18**(1): p. 1-15.
7. Aounallah-Skhiri, H., et al., *Nutrition transition among adolescents of a south-Mediterranean country: dietary patterns, association with socioeconomic factors, overweight and blood pressure. A cross-sectional study in Tunisia.* Nutr J, 2011. **10**(38): p. (24 April 2011).
8. Nasreddine, L.M., et al., *Total Usual Nutrient Intakes and Nutritional Status of United Arab Emirates Children (< 4 Years): Findings from the Feeding Infants and Toddlers Study (FITS) 2021.* Current Developments in Nutrition, 2022. **6**(5): p. nzac080.
