# Supplementary material for: Sugar Reduction Initiatives in the Eastern Mediterranean Region: A Systematic Review
Source: Nutrients. 2022 Dec 22;15(1):55. doi: 10.3390/nu15010055 (PMC9823488; doi:10.3390/nu15010055)
Supplement: Supplementary file 1 [file nutrients-15-00055-s001.zip › nutrients-2087094-supplementary/Table S6.pdf]

Table S6. Sugar Levels in Food and Meals.

Country	Reference	Year	Method used	Food categories collected	Sugar content in foods
Iran	Hadian and Mousavi Khaneghah 2021 [1]	2016	Industrial and traditional cereal-based-backed products were randomly collected from the Tehran local supermarkets and retail-markets.<br><br>Sugar content was evaluated according to the official method developed and designated by the Iranian National Standards Organization.	Industrial and traditional cereal-based-backed products [cake, sponge cake, cracker, koluche, simple biscuit, non-simple biscuit, pirashki, puffed products, and wafer, and traditional bakery products such as cake, fermented pastry (e.g., Danmarki and Napeloni), funnel pastry (e.g., Bamiyeh and Keshmeshi), Koluche, layered pastry (e.g., Baghlava), non-fermented pastry (e.g., Mikado and Zaban) and Pirashk)];<br>n=132	% sugar content:<br>- Maximum levels of industrial bakery products were found in sponge cake (29.92 ± 6.02%) followed by koluche (27.31 ± 5.07%). Minimum sugar levels were found in pirashki (17.17 ± 9.38%).<br><br>- Sugar levels in traditional bakery samples ranged from 11.12 ± 1.89% (pirashki) to 30.38 ± 13.11% (funnel pastry).
	Khoshtinat et al 2021 [2]; Cross-sectional	2017-2019	Mayonnaise and salad dressing samples were collected from the Iranian market in Tehran and evaluated according to the Iranian Institute of Standards and Industrial Research of Iran (ISIRI).	Fat-reduced mayonnaises and 11 brands of salad dressings;<br>n=12 mayonnaises and 47 salad dressings	Sugar content (g/100 g of food):<br>- Sugar levels in mayonnaise samples significantly decreased from 5.97 ± 1.14 g in 2017 to 3.63 ± 0.53 g in 2019<br>- Sugar levels of salad dressings significantly decreased from 8.97 ± 2.34 g in 2017 to 1.58 ± 2.65 g in 2019
Jordan	Aloudat et al 2020 [3]	-	Two different softwares (ESHA and NutriComp) were used.	Most popular traditional and modern meals/recipes based on the website of a famous and popular Jordanian chef, Manal Alalem;<br>n=40	Median contribution of sugar to energy:<br>3.2-3.6%
	Barham and MOH 2022, unpublished data	2022	Different brands (local and imported) of SSBs were collected from different markets.	SSBs including juices, nectars, non-carbonated soft drinks, soft drinks, energy drinks, non-alcoholic malt drinks, tea drinks and powder drinks;<br>n=33	% sugar content:<br>- Juices: 9.8-11.7%<br>- Nectars: 11.9-12.6%<br>- Non-carbonated soft drinks: 9.4-12.6%<br>- Soft drinks: 7.4-18.1%<br>- Energy drinks: 9.7-14.6%

			The collected samples were sent to Royal Scientific Society laboratory for analysis of AS levels: sucrose, fructose, lactose, maltose, glucose (dextrose).		- Non-alcoholic malt drinks: 6.7-9.2%<br>- Tea drinks: 6.3-8.5%<br>- Powder drinks: 51.7-80%
Lebanon	Hoteit et al 2021 and Hotei and Zoghbi 2021 [4, 5]	-	Stratified sampling techniques used to collect the samples; 5 strata (Mount Lebanon, Bekaa, Beirut, Tripoli, Saida).<br><br>500 g of 30 samples were collected from central kitchens in the 5 governorates; chemical analysis was performed.	Traditional Lebanese dishes among the governorates; n=150	All the traditional Lebanese dishes, in all the governorates, had little amounts of TS.<br>The highest amounts (at least 3 g/100 g of food) were as follows:<br>- In Mount Lebanon: Hindbe b zet (4.2 g) and Falafel (3.6 g)<br>- In the Bekaa: Yakhnet bemieh (5.5 g) and Lahm bi ajeen (4.7 g)<br>- In Beirut: Lahm bi ajeen (3.4 g) and Foul moudamas (3.3 g)<br>- In Tripoli: Chichbarakk (6 g) and Fattoush (5.2 g)<br>- In Saida: Falafel (4.3 g) and Lahm bi ajeen (3.2 g)
	Hoteit et al 2021 [6]	-	Recipes of traditional Arabic sweets, most frequently consumed by the Lebanese population, were converted to meal planning exchange lists.<br><br>Analysis was done using Association of Official Analytical Chemists procedures; 500 g of each was collected for analysis.	Traditional Arabic sweets from popular sweet retails;<br>n=35	<u>Number of sugar exchanges per 100 g:</u><br>- Baklava mixed; Boundoukia; Ghourayba; Halawet el Jiben; Ish el bulbul; Maamoul tamer; Maamoul mad joz; Maamoul joz; Mafrouka kashta: 1<br>- Baklava mixed light; Mouhallabiya; Riz bil halib: 0.5<br>- Maakroun and moushabbak; Moufattaka; Moushabak; Nammoura: 2<br>- Saniora: 1.25
Oman	AbuKhader 2018 [7]	2016	AS were assessed in 30 g of selected snack foods for young children from 4 to 13 years old.	Ready-to-eat cereals, chocolate confectionery and biscuits sold in supermarket outlets in Muscat, Oman; N=71	<u>AS content (g/100 g of food):</u><br>- Ready-to-eat cereals: 31.7 ± 5.2 g<br>- Chocolate confectionery: 52.2 ± 6.2 g<br>- Biscuits: 26.4 ± 9.4 g

			Sugar content was obtained from nutrition information panels.		
	Almamary and MOH 2022, unpublished data	2022	Juices, soft drinks and milk were analyzed in the lab to compare the sugar content with label	Juices, soft drinks and milk; n=43	<u>Sugar content per 100 ml:</u><br>- Juices: 0-80 g of TS and 0-53.85 g of AS<br>- Beverages: 0-4.4 g of TS and 0 g of AS<br>- Milk: 0-7.14 g of TS and 0-0.769 g of AS
Qatar	*Information provided by the NFP*	2017	The MOPH team conducted their own market research by analyzing the sugar content of SSBs.	SSBs;<br>N=140	--
	Information provided by the NFP	2016	MOPH participated in the international survey by WASSH to determine the sugar and salt content of global breakfast cereal brands.<br><br>A market search was conducted on the major grocery outlets and the breakfast cereals were analyzed for their sugar content.	Breakfast cereals	--
Tunisia	World Health Organization 2016 [8]	-	Recent analysis on several foods items; analysis included sugar content.	Processed foods, fast foods, bread and cheeses	No specific information on the findings

Abbreviations: AS: added sugars; ESHA: Elizabeth Stewart Hands and Associates; MOH: Ministry of Health; MOPH: Ministry of Public Health; NFP: nutrition focal point; SSBs: sugar-sweetened beverages; TS: total sugars; WASSH: World Action on Salt, Sugar and Health.

References

1. Hadian, Z. and A. MOUSAVI KHANEGHAH, *Sugar, fat, saturated and trans fatty acid contents in Iranian cereal-based baked products.* Food Science and Technology, 2021. **42**.
2. Khoshtinat, K., et al., *Comparative study of salt, total fat and sugar contents of mayonnaise and salad dressings from the Iranian market in 2017 and 2019.* Eastern Mediterranean Health Journal 2021.
3. Aloudat, M., et al., *NUTRITIONAL VALUE OF TRADITIONAL AND MODERN MEALS: JORDAN AND HUNGARY.* Acta Alimentaria, 2020. **49**(4): p. 491-497.
4. Al-Jawaldeh, A., et al., *Nutritional value of the Middle Eastern diet: Analysis of total sugar, salt, and iron in Lebanese traditional dishes.* F1000Research, 2020. **9**.
5. Hoteit, M. and E. Zoghbi, *Food Composition Data: Traditional Dishes, Arabic Sweets, and Market Foods*. 2021.
6. Hoteit, M., et al., *Development of a Lebanese food exchange system based on frequently consumed Eastern Mediterranean traditional dishes and Arabic sweets.* F1000Res, 2021. **10**: p. 12.
7. AbuKhader, M.M., *Examining the amounts of added sugars and saturated fatty acids recorded on the nutrition panels of snack foods for young children.* Progress in Nutrition, 2018. **20**(4): p. 570-577.
8. World Health Organization, *Summary report on the Regional meeting to standardize and update food composition tables, reflecting sugar, trans fat, saturated fat and salt contents, Rabat, Morocco, 20–22 September 2016*. 2016, World Health Organization. Regional Office for the Eastern Mediterranean.
