# Supplementary material for: Sugar Reduction Initiatives in the Eastern Mediterranean Region: A Systematic Review
Source: Nutrients. 2022 Dec 22;15(1):55. doi: 10.3390/nu15010055 (PMC9823488; doi:10.3390/nu15010055)
Supplement: Supplementary file 1 [file nutrients-15-00055-s001.zip › nutrients-2087094-supplementary/Table S7.pdf]

Table S7. Knowledge, Attitudes and Behavior (KAB) towards Sugar in countries of the EMR.

Country	Reference	Year	National or Regional	Method used	Study Population	Findings
**Bahrain**	Hussain et al 2021 [1] Cross-sectional	2017-2018	Regional	Self-administered semi-structured questionnaire	Bahraini youth aged 15-24 years; n=728	Knowledge:<br>- 7.4% cited that energy drinks cause dental caries
	Wahab 2019 [2]; Cross-sectional	2017	Regional; selected Food Market Centers	Questionnaire	Consumers; n=430	Knowledge:<br>- 55.8% of the consumers think that one of the most important nutritional items they will look at when buying a food is the sugar content of the food<br>- 30% of the consumers perceive diabetes as the most health concern
**Egypt**	Hasan Saad 2021 [3]	2021	Regional; Kfar Cheikh	Questionnaire	Adults aged 24-66 years; n=327	Behavior:<br>- 57.2% always consume a lot of sweets and juices, as compared to 15.3% sometimes<br>- 39.1% always reduce the amount of AS by a third or half, always, as compared to 37.3% sometimes<br>- 7.6% always use brown sugar or honey as sweeteners instead of white sugar, as compared to 7.6% sometimes<br>- 35.5% always substitute sugar-sweetened juices by natural juices or fruits, as compared to 22.6% sometimes<br>- 49.5% always restrict the purchase of carbonated drinks on occasions only, as compared to 23.5% sometimes<br>- 7.3% always buy sugar alternatives from pharmacies to reduce their intake of regular sugar<br>- 46.5% always avoid the purchase of large portion sizes of carbonated drinks and sweets

						even if there are offers, as compared to 3.7% sometimes
**Iran**	Ahadi et al 2014 [4]; Cross-sectional	2011-2012	National	Structured questionnaire	Households; n=14136	Behavior:<br>Sugar intake was daily in 81.6% of the households, weekly in 7.1%, rarely in 7.3% and never in 4% of the households
	Rad et al 2017 [5]; Cross-sectional	2013-2014	Regional; 5 provinces	Culturally-adapted questionnaire	Children aged 12 years; n=1554	Knowledge:<br>- 73.4% answered correctly that drinking fruit juice will prevent tooth decay<br>- 69.8% answered correctly that eating mint candies will prevent tooth decay
	Rahnama et al 2017 [6]; Cross-sectional	2012-2013	Regional; Shiraz	Pretested questionnaire	Students aged 11-15; n=2040	Knowledge:<br>- 47.9% answered the following correctly: you should eat a lot of sugar to have enough energy<br>- 54.6% do not know if high blood sugars increase cholesterol levels
	Saeidlou et al 2016 [7]; Cross-sectional	-	Regional; West Azerbaijan	Structured questionnaire	Urban and rural households; n=455	Knowledge:<br>- 33.7% were aware that high consumption of fizzy drinks may cause osteoporosis<br>- 17% were aware that high consumption of fizzy drinks may cause malnutrition<br><br>Behavior:<br>- 6.2% consumed fizzy drinks daily, 19.4% weekly, 44.9% rarely and 29.5% never<br>- 8.7% consumed chocolate daily, 26.5% weekly, 43.4% rarely and 21.4% never<br>- 86.9% consumed sugar daily, 3.8% weekly, 5.2% rarely and 4% never
	Mirmiran et al 2010 [8]; Cross-sectional	-	Regional; Tehran	Based on the Tehran Lipid and Glucose Study; validated semi-quantitative FFQ	Aged 20-70 years; n=826	Knowledge:<br>- 83.7% of males and 89.6% of females answered correctly that lower sugar intake is the best way to lose and maintain and appropriate weight

						Attitudes:<br>- 7.6% of males and 5% of females prefer sweets as snacks
	Mirmiran et al 2007 [9]	-	Regional; Tehran	Questionnaire and FFQ	Adolescents from junior high schools and high schools;<br>n=7669	Knowledge:<br>- 85% knew that drinking too many soft beverages resulted in overweight or obesity<br>- 36% did not know that sweets and candies increased weight<br><br>Behavior:<br>- 4.5% did not drink soft beverages
**Jordan**	Rajab et al 2002 [10];<br>Cross-sectional	-	Regional; urban areas	Self-administered questionnaire	Children and adolescents aged 6-16 years, and their parents;<br>n=1556	Knowledge:<br>- 80% of the parents knew about the harmful effect of sugar<br><br>Behavior:<br>- 42% of parents restricted sugar and sweets to reduce dental caries
**KSA**	Subaiea et al 2019 [11];<br>Cross-sectional	2015	National	Pre-tested questionnaire	Adolescents and adults aged 15 years and above;<br>n=783	Knowledge:<br>- Lower knowledge scores were identified in daily energy drink consumers than in 1–3 times monthly consumers
	Mumena 2021 [12];<br>Cross-sectional	-	Regional	Online survey and FFQ	Children aged 6-12 years, and their mothers;<br>n=424	Knowledge towards FS:<br>- 94.8% of mothers think that eating too much FS is bad for their child's health<br>- 34.7% think that FS is sugar added to coffee and tea, 71.2% think it is sugar added to food during processing or cooking, 45.8% think it is sugar used to prepare sweets, and 6.8% think it is the sugar that exists in fruits and milk<br>- 59.9% of mothers think that diet pepsi contains large amounts of FS; 81.1% and 86.6% correctly answered that cookies and fruit drinks, respectively, contain high amounts of FS; 96.2%

					correctly answered that plain milk does not contain high FS amounts; 59.4% correctly answered that toast bread and buns do not contain high FS amounts; only 18.4% correctly answered that strawberry flavored greek yogurt contains high FS levels<br><br><u>Maternal attitudes to limit children's intake of FS:</u><br>- 41% are trying to limit the purchase of foods that are high in FS<br>- 68.2% are trying to limit the child's intake of foods that are high in FS<br>- 92.9% are trying to provide healthy food options for their child to replace foods high in FS<br><br><u>Maternal behavior to limit children's intake of FS:</u><br>- 15.1% always read the nutrition fact label of their child's favorite products to determine the amount of FS intake; 66.7% sometimes and 18.2% never<br>- 93.4% are discussing with their child the importance of replacing foods high in FS with healthy food options<br>- Only 1.7% were successful in limiting/controlling their child's FS intake
Mumena et al 2020 [13];<br>Cross-sectional	2019	Regional; Madinah	Questionnaire	Female undergraduate students at Taibah University;<br>n=190	<u>Knowledge:</u><br>- 58.8% were aware of the health consequences of excessive intakes of AS<br><br><u>Behavior:</u><br>- 32.6% were making an effort to reduce their AS intake
Hakim 2020 [14]	2017-2018	Regional; Jeddah	Questionnaire	Females aged 18 years and above from private universities;	<u>Knowledge:</u><br>- 83.6% answered that eating high amounts of sugar can cause diabetes

				n=141	
Sultan et al 2020 [15]; Cross-sectional	2017-2018	Regional; Abha	Self-administered questionnaire	Female college students at King Khalid University; n=300	<u>Knowledge:</u><br>74% knew that energy drinks contain sugar
Alshammary et al 2019 [16]; Cross-sectional	-	Regional	Questionnaire	Parents; n=223	<u>Knowledge:</u><br>Almost 97% of parents knew that sugar and sticky food had a bad effect on oral health
Alrasheedi 2016 [17]	2014	Regional; Jeddah	Self-administered questionnaire	Students age 12-26 years at intermediate and high schools and universities; n=4355	<u>Knowledge:</u><br>86-91.5% of the students think that energy drinks contain sugar
Quadri et al 2015 [18]; Cross-sectional	-	Regional; Jazan	Questionnaire	Schoolchildren aged 6-15 years; n=853	<u>Knowledge:</u><br>-71.7% of males and 72.9% of females had poor knowledge in distinguishing between various forms of sweet foods<br><br>-65.9% of males and 66.9% of females did not know which drinks contained more sweeteners<br><br>-56.1% of males and 70.8% of females thought soft drinks were healthier than fruit juice<br><br><u>Attitudes:</u><br>The percentage of males and females who preferred soft drinks to mineral water were 83.5% and 85.8% respectively.<br><br>- 94.2% of males and 93.4% of females preferred sweets to plain bread<br>- 86.6% of males and 96.1% of females did not prefer eating snacks with less sugar

						Behavior:<br>- Among both males and females, > 90% of the children reported that they frequently consumed soft drinks, sweets, milk with sugar and chocolate
	Aluqmany et al 2013 [19];<br>Cross-sectional	-	Regional; Almadinah Almunawwarah	Interview questionnaire	Secondary school female students;<br>n=600	Knowledge:<br>- 77.3% believe that energy drinks have an effect on blood sugar
	Wyne et al 2004 [20]	-	Regional; Riyadh	Self-administered questionnaire	Male secondary school children;<br>n=605	Knowledge:<br>- 94.5% knew that sweets (chocolates/candies) could cause tooth decay<br>- 40.2% were not aware of cariogenic potential of soft drinks/bottled juices<br>- 77.5% were not aware of cariogenic potential of sweetened milk
	Wyne et al 2002 [21]	-	Regional; Riyadh	Self-administered questionnaire	Teachers from schools;<br>n=39	Knowledge:<br>- 97.4 % understood the main cause of dental caries which is high sugar intake<br>-28.2% did not think that frequent intake of soft drinks was a caries risk factor
	Al-Othaimeen et al 1999 [22]	-	Regional; Riyadh	Questionnaire	Female students from primary schools;<br>n=1210	Behavior:<br>16.5% depend on snacks offered in school canteens; mainly biscuits, chocolate bars and carbonated cola drinks
**Kuwait**	MOH [23]	2014	National	STEPs Questionnaire	Adults aged 18-69 years	Behavior:<br>Number of sugar spoons added to each cup of hot and cold beverages:<br>- 28.1% add 1 teaspoon<br>- 39.8% add 2 teaspoons<br>- 5.3% add 3 or more teaspoons<br>- 26.8% do not add any
**Lebanon**	Hoteit et al 2022 [24];<br>Cross-sectional	2020	National	Questionnaire	Adolescents and adults aged 15-64 years;<br>n=768	Knowledge:<br>- 48.6% knew what a “sugar-free” claim indicates

						Attitudes:<br>- 66.4% expressed positive attitudes to check information related to sugars<br><br>Behavior:<br>- 44.3% reported reading labels related primarily to sugars
	Rahman 2016 [25];<br>Mixed methods	-	Regional; Phase 1 in Pediatric Dental Clinic at the Lebanese University and Phase 2 in in Greater Beirut area	24-hr recall	Phase 1:<br>Mothers of children aged 4-7 years;<br>n=30<br><br>Phase 2:<br>Children aged 9-12 years attending public schools;<br>n=143	Phase 1-Behavior:<br>- 80% of mothers who provided milk to their children before going to school, added sugar to it<br>- Around 50% of mother reported that their children bought juice from the school shop<br>- Around 1/3rd of the mothers offered soft drinks to their children during weekends<br><br>Phase 2-Behavior:<br>- 91% of children who consumed milk, added sugar to it<br>- 79% of children who consumed tea, added sugar to it<br>- During lunch at school, 21% consumed soda drinks<br>- With the snack at school, 34% drank juice
**Morocco**	MOH [26]	2017	National	STEPs Questionnaire	Adults aged 18 years and above	Behavior:<br>Number of sugar spoons added to hot milks:<br>- 37.4% add 1 spoon<br>- 38% add 2 spoons<br>- 17.1% add 3 spoons<br>- 7.6% do not add any
	Chalgoum et al 2009 [27];<br>Retrospective	2007	Regional; Zaghouan	Questionnaire	Children aged 3-5 years from 4 kindergartens;<br>n=160	Behavior:<br>Food behavior showed consumption of diets high in sugar (87.5%)
**Oman**	Manickavasagan et al 2014 [28]	-	Regional; Muscat	Questionnaire	Students from 10 colleges;	Knowledge:

					n=1191	39% were aware of the unhealthy effects of high sugar consumption
	Al Riyami et al 2010 [29]; Cross-sectional	2005	Regional; Nizwa	Structured questionnaire	Elderly people aged 60 years and above; n=2041	Knowledge:<br>- 22.3% (21.3% males and 23.2% females) believe that sweets/sugar should be consumed in moderation<br><br>Behavior:<br>- 10.9% (9.6% males and 12% females) restrict sugar in their diet regimen
**Pakistan**	Ministry of National Health Services Regulations and Coordination 2021 [30]	2021	Regional; Islamabad and in 4 provinces	Survey (via the phone and face-to-face interviews)	Adults; n=6824	Knowledge:<br>- 88% were unable to identify the correct quantity of sugar in a soda bottle<br>- 81.3% think that SSBs are a main reason of obesity in Pakistan<br>- 84% think that SSBs are a source of daily intake of unnecessary sugars<br>- 87.2% think that SSBs cause serious health problems<br><br>Attitudes:<br>- 85% believe the government should take steps to discourage the consumption of sugary drinks<br>- Approximately 78% support implementing taxes on SSBs<br><br>Behavior:<br>- 29% consumed SSBs weekly and 17% on a daily basis (9.2% packaged juice and 7.8% soda drink)
	Luqman et al 2021 [31]; Transverse	-	Regional; 3 districts of Southern Punjab	Questionnaire	Population; n=600<br><br>1: strong disagree<br>2: disagree	Knowledge:<br>- Mean agreement level that sugar, sweets and beverages (Barfi, Honey, Jalebi, Sugar, Gur and Carbonated beverages) are good micro and macronutrients sources for health: 3.07 ± 1.09

				3: neutral<br>4: agree<br>5: strongly agree	among rural people and 2.58 ± 1.02 among urban people<br><br><u>Behavior:</u><br>- Mean agreement level to replace one sugary drink (soda, juice, etc.) each day with a tall glass of water: 3.27 ± 1.033 among rural people and 3.62 ± 1.013 among urban people
Tanvir et al 2018 [32]; Cross-sectional	2016	Regional; Rawalpindi cantonment	Questionnaire	Adults aged 18 years and above; n=100	<u>Knowledge:</u><br>- 15% were aware that sweets are a risk factor for cardiac disease
Rashid et al 2018 [33]; Cross-sectional	2013-2014	Regional; Azad Kashmir	Pretested questionnaire	Secondary school students; n=384	<u>Knowledge:</u><br>- 98.7% answered correctly that too much sweet food causes tooth decay/dental caries<br><br><u>Behavior:</u><br>- 59.1% eat sweet foods 2-4 times/day (9.9% among males and 29.2% among females)<br>- 17.5% eat sweet foods more than 4 times/day (8.1% among males and 9.4% among females)<br>- 23.9% have soft drinks 2-5 times/week (10.7% among males and 13.3% among females)<br>- 49% have soft drinks everyday (21.1% among males and 27.9% among females)
Sayed 2012 [34]; Cross-sectional	2011	Regional; Karachi	Self-administered questionnaire	University students aged 15-30 years in various institutes; n=260	<u>Behavior:</u><br>- 83.4% were having cakes and biscuits daily<br>- 79% consumed soft drinks daily<br>- 58% were aware of recommendation to reduce sugar<br>- 41.9% are not sugar if raw sugar is better than white sugar, while 28.1% and 11.5% agree and strongly agree, respectively

Zuhaid et al 2012 [35]; Cross-sectional	2010	Regional; seven different localities of Peshawar	Questionnaire	Residents aged 15-60 years; n=305	Knowledge:<br>- 46.2% of the subjects identified excessive sugar intake as a major risk factor of diabetes

Abbreviations: AS: added sugars; EMR: Eastern Mediterranean Region; FFQ: food frequency questionnaire; FS: free sugars; KAB: knowledge, attitudes and behaviors; MOH: Ministry of Health; SSBs: sugar-sweetened beverages.

References

1. Hussain M, Perna S, Mandeel Q, Naser J, Alalwan T. Awareness and Consumption Pattern of Energy Drinks Among Bahraini Youth. Bahrain Med Bull. 2021;43(2).
2. Wahab RA. Food Label Use and Awareness of Nutritional Information Among Consumers in Bahrain: An Exploratory Study. KnE Life Sciences. 2018:26-36.
3. Hasan Saad F. الإستخدام الآمن للريفيات من الملح والسكر والدهون ببعض قرى محافظة كفرالشيخ. Agric Econ Soc Sci. 2021;12(8):669-81.
4. Ahadi Z, Heshmat R, Sanaei M, Shafiee G, Ghaderpanahi M, Homami MR, et al. Knowledge, attitude and practice of urban and rural households towards principles of nutrition in Iran: Results of NUTRIKAP survey. J Diabetes Metabolic Disord. 2014;13(1).
5. Rad M, Shahravan A, Haghdoost AA. The dietary patterns of 12-year-old children and their awareness about the effect of diet on oral health in Iran. J Oral Health Oral Epidemiol. 2017;6(2):102-9.
6. Rahnama R, Rampal L, Lye MS, Mohd. Sidik S, Abedi P. Diet knowledge and behaviors related to prevention of obesity among students aged 11 to 15 years in Shiraz, Iran. Iran Red Crescent MedJ. 2017;19(8).
7. Saeidlou SN, Babaei F, Ayremlou P. Nutritional Knowledge, Attitude and Practice of North West Households in Iran: Is Knowledge likely to Become Practice? Maedica (Bucur). 2016;11(4):286-95.
8. Mirmiran P, Hosseini-Esfahani F, Jessri M, Mahan LK, Shiva N, Azizi F. Does Dietary Intake by Tehranian Adults Align with the 2005 Dietary Guidelines for Americans? Observations from the Tehran Lipid and Glucose Study. J Heatlh Popul Nutr. 2011;29(1):39-52.
9. Mirmiran P, Azadbakht L, Azizi F. Dietary behaviour of Tehranian adolescents does not accord with their nutritional knowledge. Public Health Nutr. 2007;10(9):897-901.
10. Rajab LD, Petersen PE, Bakaeen G, Hamdan MA. Oral health behaviour of schoolchildren and parents in Jordan. Int J Paediatr Dent. 2002;12(3):168-76.
11. Gehad MS, Ali FA, Thamir MA. Energy drinks and population health: consumption pattern and adverse effects among Saudi population. BMC Public Health. 2019;19(1):1539-.
12. Mumena WA. Maternal Knowledge, Attitude and Practices toward Free Sugar and the Associations with Free Sugar Intake in Children. Nutr. 2021;13(12):4403.
13. Mumena WA, Alamri AA, Mahrous AA, Alharbi BM, Almohaimeed JS, Hakeem MI, et al. Knowledge, attitudes, and practices toward added sugar consumption among female undergraduate students in Madinah, Saudi Arabia: A cross-sectional study. Nutrition. 2020;79-80:6.
14. Hakim NA. Diabetes awareness and dietary habits of non-diabetic females in private universities in Jeddah city; Saudi Arabia. Prog Nutr. 2020;22(4):9.
15. Sultan AA, Al Saleem SA, Osman AAM, Mostafa OA. Knowledge and Prevalence of Energy Drinks Consumption among King Khalid University Female Students. World Fam Med. 2020;18(4):4-17.
16. Alshammary F, Aljohani FA, Alkhuwayr FS, Siddiqui AA. Measurement of parents' knowledge toward oral health of their children: An observational study from Hail, Saudi Arabia. J Contemp Dental Pract. 2019;20(7):801-5.
17. Alrasheedi AAJGJHS. Prevalence and reasons for consumption of energy drinks among adolescents and young adults in Jeddah, Saudi Arabia. 2016;9:23-8.

18. Quadri FA, Hendriyani H, Pramono A, Jafer M. Knowledge, attitudes and practices of sweet food and beverage consumption and its association with dental caries among schoolchildren in Jazan, Saudi Arabia. East Mediterr Health J. 2015;21(6):403-11.
19. Aluqmany R, Mansoor R, Saad U, Abdullah R, Ahamd A. Consumption of energy drinks among female secondary school students, Almadinah Almunawwarah, Kingdom of Saudi Arabia, 2011. J Taibah Univ Med Soc. 2013;8(1):60-5.
20. Wyne AH, Chohan AN, Al-Dosari K, Al-Dokheil M. Oral health knowledge and sources of information among male Saudi school children. Odontostomatol Trop. 2004;27(106):22-6.
21. Wyne AH, Al-Ghorabi BM, Al-Asiri YA, Khan NB. Caries prevalence in Saudi primary schoolchildren of Riyadh and their teachers' oral health knowledge, attitude and practices. Saudi Med J. 2002;23(1):77-81.
22. Al-Othaimeen A, Osman AK, Al Orf S. Prevalence of nutritional anaemia among primary school girls in Riyadh City, Saudi Arabia. Int J Food Sci Nutr. 1999;50(4):237-43.
23. Ministry of Health-Kuwait. Eastern mediterranean approach for control of non communicable diseases. Survey for risk factors for chronic non communicable diseases. 2015.
24. Hoteit M, Yazbeck N, Al-Jawaldeh A, Obeid C, Fattah HA, Ghader M, et al. Assessment of the knowledge, attitudes and practices of Lebanese shoppers towards food labeling: The first steps in the Nutri-score roadmap. F1000 Res. 2022;11.
25. Rahman AA. A theory-based exploration of adding sugar to beverages in Lebanese public school children: University of Strathclyde; 2016.
26. Ministry of Health-Morocco. Enquête Nationale sur les Facteurs de Risque communs des Maladies Non Transmissibles 2017 – 2018: Rapport. 2017.
27. Chalgoum NB, Koubaa AA, Dahmen H, Kochbati A. Nutritional practices of young children. Tunis Med. 2009;87(11).
28. Manickavasagan A, Al-Mahdouri AA, Al-Mufargi AMS, Al-Souti A, Al-Mezeini ASM, Essa MM. Healthy eating knowledge among college students in Muscat: A self reported survey. Pak J Nutr. 2014;13(7):397-403.
29. Al Riyami A, Al Hadabi S, Abd El Aty MA, Al Kharusi H, Morsi M, Jaju S. Nutrition knowledge, beliefs and dietary habits among elderly people in Nizwa, Oman: Implications for policy. East Mediterr Health J. 2010;16(8):859-67.
30. Ministry of National Health Services Regulations and Coordination-Pakistan. National Views on Sugar Sweetened Beverages in Pakistan - 2021 Opinion Poll. Pakistan: Pakistan Health Research Council-Ministry of National Health Services, Regulations and Coordination; 2021.
31. Luqman M, Hussain R, Yaseen M, Mehmood MU, Asghar I, Saleem U. Comparative Analysis of Dietary Intake Patterns of Rural and Urban Communities of Southern Punjab, Pakistan. Sarhad J Agri. 2021;37(1):128-35.
32. Tanvir S, Sajjad S, Roshan R. Awareness levels of prevention of cardiac diseases in general population of rawalpindi and requirement of health education. Public Health. 2018;157:107-10.
33. Rashid M, Saeeda R, Mudassar I. Knowledge, attitude and practice regarding oral health among secondary school students of Azad Kashmir, Pakistan. Pakistan Journal of Public Health. 2018;8(1):32-6.
34. Sayed SA. A cross-sectional study on eating habits and food related beliefs and knowledge in university students of Karachi, Pakistan. Pakistan Journal of Public Health. 2012;2(2):36-42.
35. Zuhaid M, Zahir KK, Diju IU. Knowledge and perceptions of diabetes in urban and semi urban population of Peshawar, Pakistan. J Ayub Med Coll Abbottabad. 2012;24(1):105-8.
